# Mamba-based state-space modeling with window attention for explainable multi-class gastrointestinal disease diagnosis in endoscopic images

**DOI:** 10.3389/frai.2026.1952159

**Published:** 2026-09-17

**Authors:** Vishesh Tanwar, Bhisham Sharma, Julian L. Webber, Abolfazl Mehbodniya

**Affiliations:** 1 Chitkara University Institute of Engineering and Technology, Chitkara University, Rajpura, Punjab, India; 2 Department of Electronics and Communication Engineering, Kuwait College of Science and Technology, Doha Area, Kuwait

**Keywords:** diagnostic, endoscopic image analysis, explainable artificial intelligence, gastrointestinal disease classification, hybrid deep learning, medical image classification, state-space model (mamba), window attention

## Abstract

**Introduction:**

This paper introduces a new hybrid deep learning architecture, which is named GINet, to accurately and efficiently classify gastrointestinal (GI) diseases using endoscopic images. Automated diagnosis is not an easy task due to the visual similarity among GI conditions and the class imbalance.

**Method:**

To overcome these challenges, the proposed model is based on EfficientNetV2 for powerful feature extraction, supported by a window-based attention mechanism to capture fine-grained local spatial dependencies. Moreover, a state-space module based on Mamba is added to model long-range global contextual relationships with linear computational complexity. It presents a complete data preprocessing pipeline, including class filtering, imbalance management via augmentation, and a leakage-free train-test split. The dataset consists of nine clinically relevant GI classes, with equal distribution for training. The extracted features are further refined by a channel projection layer, and the combined Window Attention and Mamba modules enable the model to learn local and global representations.

**Results:**

Extensive experiments show that the proposed GINet achieves 99.41% validation accuracy after classifier fine-tuning and 93% accuracy on the held-out test set, and high precision, recall, and F1 scores across all classes. Statistical analysis shows that the gains in performance are significant compared to the baseline models. Also, explainable artificial intelligence, as demonstrated by Grad-CAM, shows that the model attends to clinically meaningful regions, promoting transparency and trust.

**Discussion:**

The suggested solution offers a computationally efficient, accurate and interpretable solution to computer-aided diagnosis of gastrointestinal diseases, and represents a step toward computer-aided diagnosis of gastrointestinal diseases, subject to further validation on external, multi-center data.

## 1 Introduction

Gastrointestinal (GI) diseases are a major global health problem that require early, accurate diagnosis to enhance patient outcomes. Manual analysis of endoscopic images is a time-consuming, subjective, and extremely clinician dependent method of endoscopy surveillance, but is still the gold standard of endoscopy surveillance (Vania et al., 2023). With the increasing volume of endoscopic procedures, there is a growing need for automated, reliable, and scalable computer-aided diagnostic (CAD) systems. Recent developments in deep learning, specifically Convolutional Neural Networks (CNNs) have demonstrated impressive results in the analysis of medical images (Ali et al., 2020). Other architectures like EfficientNet have shown high extraction of features with optimal parameter efficiency. Nonetheless, traditional CNNs typically have difficulty in capturing long-range dependencies and fine-grained spatial relationships, which is important in differentiating visually similar GI conditions (Naz et al., 2021). However, these problems could be addressed using attention control techniques to enhance feature representation capability. One effective technique is the Window Attention that has been proven to be an efficient approach to modeling local spatial dependencies in computation-friendly ways. Localization of attention, compared with global self-attention, a computationally inefficient method, allows for capturing localized or regional areas in order to extract more discriminative features like mucosal texture, vascular structures and lesion boundaries, all of which serve as important factors in the diagnosis of GI diseases (Min et al., 2019).

As they mainly concentrate on extracting local spatial features and usually fail to learn long-range contextual dependencies that exist in the endoscopic images, conventional CNN-based models have a lot of disadvantages in the category of GI diseases (Kuntz et al., 2021). It is difficult to discriminate accurately between classes for conventional CNNs since there are slight texture differences in GI diseases. Moreover, class imbalance is another issue, which causes learning bias toward dominant classes since some problematic classes have far fewer training samples than others. Another drawback is the lack of transparency and poor predictability of conventional CNN-based models, especially for noisy endoscopic frames, motion blur, complicated mucosa structures and variations in illumination conditions (Tanwar et al., 2025). Furthermore, although they achieve competitive accuracy, they cannot perform well in various datasets without extensive feature engineering and heavy computational costs.

Despite the improvement in feature representations made by hybrid CNN-Transformer models, current hybrid frameworks still have some problems. Some hybrid frameworks are computationally inefficient because of integrating two or even more backbone networks and attention models, which inevitably results in high training costs, excessive memory consumption and complicated inferencing process (Zhuang et al., 2023). Also, feature redundancy occurs for some hybrid models as a result of difficulty in finding an optimal balance between local and global information. Misclassification will occur in gastrointestinal image classification when lesions are visually similar and/or share overlapping anatomy in hybrid models.

Additionally, most existing hybrid models rely heavily on large annotated datasets and may exhibit reduced performance on highly imbalanced or clinically diverse datasets (Aadiwal et al., 2025). Another major limitation is the lack of robust explainability and progression-aware learning in many frameworks, reducing their reliability for real-world clinical decision support. These limitations motivate the development of more efficient, lightweight, explainable, and context-aware transformer-based architectures for accurate gastrointestinal disease classification (He et al., 2025).

In this research, we introduce a new deep learning architecture that uses EfficientNetV2 to efficiently extract features and a Window Attention mechanism to better understand the space. The model pipeline starts with data augmentation methods, such as random rotation, resized cropping, color jittering, and horizontal flipping, to enhance generalization and address class imbalance. The extracted features are refined by a channel projection layer (1×1 convolution) into a feature that captures fine-grained local dependencies (Window Attention module). These refined feature maps are then passed through Global Average Pooling (GAP) layer to reduce the dimensionality and are then fully connected layers with dropout regularization to classify. The proposed model categorizes nine GI tract conditions: Accessory tools, Duodenal bulb, Dyed-resection margins, Gastroesophageal junction (normal Z-line), Ileocecal valve, Normal mucosa and vascular pattern in the large bowel, Normal stomach, Pylorus, and Small bowel (terminal ileum).

The key contributions of this work are as follows:

- A novel hybrid architecture (GINet) integrating EfficientNetV2, Window Attention, and Mamba for joint local–global feature learning in GI image classification.
- An efficient global context modeling strategy using Mamba, reducing computational complexity compared to conventional self-attention.
- A feature refinement mechanism via 1 × 1 channel projection to enhance discriminative representation with minimal overhead.
- A robust dataset preprocessing pipeline including class filtering, augmentation-based balancing, and leakage-free data splitting.
- A lightweight fine-tuning strategy that updates only the classifier head, reducing training cost while improving performance. Comprehensive evaluation with statistical validation (paired t-test and Wilcoxon test) confirming significant performance gains.

In addition to this, the unique feature of the proposed model lies in its combination of local and global features via window attention and Mamba, which, to the best of our knowledge, has not previously been reported for gastrointestinal image classification. Moreover, another improvement is that the global self-attention operation has been substituted with a much more efficient state space model known as Mamba. Moreover, in addition to the above-mentioned improvements, the suggested experiment also includes an appropriate balance of the dataset and statistically sound analysis of results to deliver an accurate yet efficient and clinically meaningful framework.

This paper is divided into various sections to systematically provide a description of the suggested gastrointestinal disease classification approach. Section one of this paper discusses the significance and difficulties in gastrointestinal disease diagnoses and discusses the involvement of deep learning algorithms in performing automated analysis. Section two of this paper provides an overview of existing literature on GI image classifications, attention-based approaches, and hybrid deep learning algorithms. Section three talks about the methodology adopted in the study, which includes data acquisition, data pre-processing, augmentation, model development, training, and assessment. Section four covers experimental results, analysis, and comparisons of our model with other existing approaches. In the fifth section, talk about the ablation study performed in order to evaluate the contributions of the different components of the model to its success. Section Six discusses the clinical implications, limitations of the study, and directions for future research. Finally, the manuscript is concluded in the last section.

## 2 Related work

Recent developments in deep learning have greatly enhanced automated diagnosis of gastrointestinal (GI) diseases based on endoscopic images. The initial strategies mainly depended on the traditional convolutional neural networks (CNNs) and the transfer learning methods.

He et al. (2025) has proposed DivGI, a single deep learning system for classifying gastrointestinal endoscopy images that addresses three key challenges in GI datasets: class imbalance, indistinct lesion characteristics and high inter-class similarity. Also, the hierarchical joint label learning enhanced the capability of the model to express the relationships among GI classes. The experimental assessment of the HyperKvasir, Upper GI, and GastroVision datasets showed better results, with Matthews Correlation Coefficient (MCC) scores of 91.31, 86.72, and 82.88, respectively.

Krenzer et al. (2026) explored the issue of excessively high or excessively low-class imbalance in gastroscopy image classification with a deep learning-based medical imaging pipeline. The authors created a curated dataset consisting of four categories of clinical significance: normal, esophagitis, Barrett’s esophagus, and gastric polyps to conduct the analysis. The dataset was constructed by mixing and rearranging samples of the HyperKvasir and GastroVision datasets, creating a highly skewed distribution that is very similar to the real-world diagnostic situation. The experimental results showed that the suggested imbalance-reduction strategies led to a considerable increase in the overall classification performance, and, in particular, the minority gastric polyp classification. The highest performing model recorded an improvement of 40.4% in the F1-score as compared to the baseline approach.

Pan et al. (2025) investigated the use of artificial intelligence to classify gastrointestinal endoscopic images to enhance AI-assisted diagnostic systems. Two deep learning models, DenseNet-121 and ResNet-152, were studied on the GastroVision and Kvasir gastrointestinal endoscopy 16,220 image datasets. On small-scale classification problems, the models achieved classification accuracies of up to 97% whereas multi-class classification with 12 classes achieved an accuracy of 84%. Moreover, it was shown to be scalable to a complex gastrointestinal diagnostic scenario, as the framework was expanded to 19 categories with encouraging outcomes.

Nath et al. (2024) suggested a deep learning-based system of automated classification of gastrointestinal polyps on endoscopic images to facilitate early diagnosis of colorectal cancer. The proposed model showed a good classification performance with training, validation, and testing accuracies of 91.99, 92.29 and 89.00, respectively. Moreover, the framework was highly specific, precise, and high F1-score, meaning that it successfully differentiated between polyp types. The research also proposed a user interface as a component of a clinical decision support system to help healthcare workers to choose and classify endoscopic images.

Kakarla et al. (2026) came up with a Vision Transformer-based system to detect multiple classes of gastrointestinal diseases in endoscopic images. This model was tested on the GastroVision dataset containing 8,600 endoscopic images in 27 gastrointestinal disease classes. The results of the experiments proved that the proposed Vision Transformer-based model achieved a recall of 85.75, precision of 86.41, and F1-score of 85.57. Moreover, the proposed method was more successful than the DenseNet-121 architecture, which means that the use of transformer-based architectures is effective to complete the large-scale gastrointestinal disease classification tasks.

Subedi et al. (2025) suggested a hybrid CNN-Transformer architecture to classify endoscopy and video capsule endoscopy images to enhance the early detection of gastrointestinal cancer and anomalies. The features that were extracted in both arms were combined to carry out powerful gastrointestinal image classification. The suggested model was tested on the GastroVision and Kvasir-Capsule datasets. On the GastroVision dataset, the framework obtained a precision of 0.8320, recall of 0.8386, F1-score of 0.8324, accuracy of 0.8386 and Matthews Correlation Coefficient (MCC) of 0.8191, outperforming conventional CNN models and standalone Swin Transformer architectures and effectively handling class imbalance. On the Kvasir-Capsule dataset, likewise, the model had better performance, with an accuracy of 0.7239 and it was observed that the model was better at classification than the existing methods.

Boppana et al. (2026) proposed a framework known as GastroMalign, which is based on a vision transformer and is developed to detect early gastrointestinal lesions and stratify based on malignancy-risk risk factors on a case-by-case basis using endoscopic images. The proposed framework, in contrast to the conventional artificial intelligence systems that mainly involve binary classification, involved progression-aware ordinal risk assessment to categorize lesions on a benign-to-malignant spectrum. The framework was tested on the publicly available GastroVision dataset of 8,000 endoscopic images of various regions of the gastrointestinal tract, such as esophagus, stomach, duodenum, colon, rectum, and terminal ileum. Experimental results showed that GastroMalign had a overall accuracy of 80.06, precision of 79.65, recall of 80.06 and F1-score of 79.17, with a micro-averaged AUC of 0.98 on the held-out test set. The proposed framework significantly performed better than baseline CNN models, including ResNet-50 and DenseNet-121, and also the Swin Transformer model. Ablation analysis also showed how critical the progression-aware module is because when it is removed the high-risk lesion recall is reduced by 17%. Furthermore, Score-CAM visualizations were found to have a 92% overlap with the regions of lesions annotated by clinicians, which demonstrates the interpretability and clinical reliability of the framework.

Tabassum et al. (2025) introduced a Vision Transformer-based endoscopic images ensemble learning model, called GastroViT. The suggested structure involved a collection of two pre-trained Vision Transformer networks, MobileViT_XS and MobileViT_V2_200, to label gastrointestinal diseases using the publicly accessible HyperKvasir dataset of 10,662 gastrointestinal disease images in 23 disease categories. In the 23-class classification task, individual models were found to have an accuracy of 90.57 and 90.48%, respectively, while the ensemble-based GastroViT model achieved an overall accuracy of 91.98%. In the first assessment, using 23 classes, GastroViT obtained average precision, recall and F1-score values of 69, 63, and 64, respectively, but with a rather lightweight architecture with about 20 million parameters and without data augmentation despite severe dataset imbalance. The framework performed even better in the second assessment that used 16 classes, and it obtained values of 87, 86, 87, and 92.70% in terms of precision, recall, F1-score, and accuracy, respectively.

Boppana et al. (2025) to build the dataset in a time-efficient manner, 15 representative frames in each video were sampled using K-means clustering applied to the feature embeddings produced by a pretrained Vision Transformer model. Expert endoscopists manually coded the resulting 5,296 frames into 29 gastrointestinal diagnostic categories, which included pathological abnormalities, such as polyps and ulcers, anatomical landmarks, and classes of mucosal quality assessment. The experimental results showed that the proposed model performed well with an overall accuracy of 90.0 percent in classifying data on the held-out test set. Moreover, the framework achieved Top-3 and Top-5 accuracies at 98.2% and 99.0, respectively, which implies that the diagnostic prediction capability is highly reliable. The weighted F1-score of 0.89 supported balanced performance in all 29 diagnostic categories. Further analysis of receiver operating characteristic analysis revealed that area under the curve values exceeded 0.98 in 25 out of 29 classes, indicating that the receiver operating characteristic analysis has excellent capability to classify summary of the literature is shown in Table 1.

**Table 1:** Summary of literature survey.

Ref.	Method/model	Dataset	Key contribution	Performance
He et al. (2025)	DivGI framework with balanced sampling, fine-grained learning, and hierarchical multi-label classification	HyperKvasir, Upper GI, GastroVision	Addressed class imbalance and inter-class similarity in GI image classification	MCC: 91.31% (HyperKvasir), 86.72% (Upper GI), 82.88% (GastroVision)
Krenzer et al. (2026)	CNN and Transformer models with Focal Loss and Cost-Sensitive Learning	HyperKvasir + GastroVision	Solved extreme class imbalance in gastroscopy image classification	40.4% improvement in minority-class F1-score
Pan et al. (2025)	DenseNet-121 and ResNet-152	GastroVision, Kvasir	AI-based GI diagnostic framework for multi-class classification	97% accuracy (small-scale), 84% accuracy (12-class)
Nath et al. (2024)	ResNet50 transfer learning	GastroVision	Automated GI polyp classification and clinical decision support system	Test Accuracy: 89%, Precision: 97.16%, F1-score: 81.65%
Kakarla et al. (2026)	Vision Transformer (ViT)	GastroVision	Multi-class GI disease detection using transformer architecture	Precision: 86.41%, Recall: 85.75%, F1-score: 85.57%
Subedi et al. (2025)	Hybrid DenseNet201 + Swin Transformer	GastroVision, Kvasir-Capsule	Combined local and global feature learning for GI anomaly detection	Accuracy: 83.86%, MCC: 0.8191
Boppana et al. (2026)	GastroMalign (ViT + Sequential Feature Learner)	GastroVision	Ordinal malignancy-risk stratification with explainability	Accuracy: 80.06%, AUC: 0.98
Tabassum et al. (2025)	GastroViT ensemble (MobileViT_XS + MobileViT_V2_200) with Grad-CAM & SHAP	HyperKvasir	Ensemble transformer learning with explainable AI	Accuracy: 91.98% (23-class), 92.70% (16-class)
Boppana et al. (2025)	Fine-tuned Vision Transformer	Clinical endoscopy dataset (5,296 images, 29 classes)	Automated GI endoscopic finding classification using ViT	Accuracy: 90.0%, Top-5 Accuracy: 90.0%, Weighted F1-score: 0.89

The first is CNN-attention hybrids, which augment a convolutional backbone with channel- or spatial-attention modules; a recent example integrates Inception, Residual, Attention, and Global Context modules and compares five attention mechanisms for multiclass GI disease classification Hosny et al. (2026) a related lightweight variant from the same group applies multiscale attention to a MobileNet backbone for the same task (Hosny and Elgendy, 2026). The second is CNN-Transformer hybrids, such as the DenseNet201 + Swin Transformer pipeline of Subedi et al. (2025) and the CNN-Transformer pipeline of Krenzer et al. (2026), which rely on self-attention to model global context; because self-attention cost scales quadratically with the number of spatial tokens, these designs trade classification performance for higher memory and compute overhead. The third family is pure vision state-space backbones, such as Vision Mamba Zhu et al. (2024) and V Mamba Liu et al. (2024), which replace convolution and self-attention entirely with stacked selective state-space (SSM) blocks built on the original Mamba formulation Gu and Dao (2023). The proposed GINet differs from all three families in how it combines, rather than replaces, these mechanisms: it retains a compact EfficientNetV2-S convolutional backbone for local texture extraction, applies Window Attention only within local, non-overlapping regions rather than globally, and reserves a single Mamba-based selective state-space block specifically to propagate long-range context across the windowed feature map after local attention has already been computed. This backbone-then-window-attention-then-SSM ordering preserves the linear-complexity advantage of SSMs for global context while avoiding both the quadratic cost of full self-attention used in CNN-Transformer hybrids and the loss of fine-grained local convolutional detail seen in attention-only or SSM-only backbones.

## 3 Materials and methods

The proposed framework presents an efficient deep learning architecture for automated classification of gastrointestinal (GI) diseases using endoscopic images, as shown in Figure 1. The pipeline begins with data collection and preprocessing, where images are resized and normalized to maintain consistency. To address class imbalance and enhance model generalization, a comprehensive data augmentation strategy is applied, including random rotation, random resized cropping, color jittering, and horizontal flipping. All these provide for a varied and well-balanced dataset. To perform feature extraction, EfficientNetV2 is used as the backbone network. This is an effective network that efficiently exploits both low-level information (texture) and high-level representations (semantic features) from complex images, such as endoscopic images. The feature maps generated by EfficientNetV2 pass through a channel projection layer with a 1 × 1 convolution to be refined and redundant. Moreover, Window Attention is implemented as an additional mechanism aimed at improving the process of learning new representations. The technique implies splitting the feature maps into smaller patches (windows) to calculate self-attention in each patch separately. As a result, it becomes possible to highlight localized spatial dependencies that are necessary for capturing such fine details of GI abnormalities as mucosal patterns, vessel structures, and lesions’ borders. Then, the global average pooling is applied to decrease the size and get more discriminative features. The extracted features undergo a classification head composed of a flatten layer, dropout, and dense layers followed by a softmax activation that outputs the probability scores of all 9 classes. In the course of training, categorical cross-entropy loss is minimized using adaptive optimization algorithms, like Adam.

**Figure 1:**
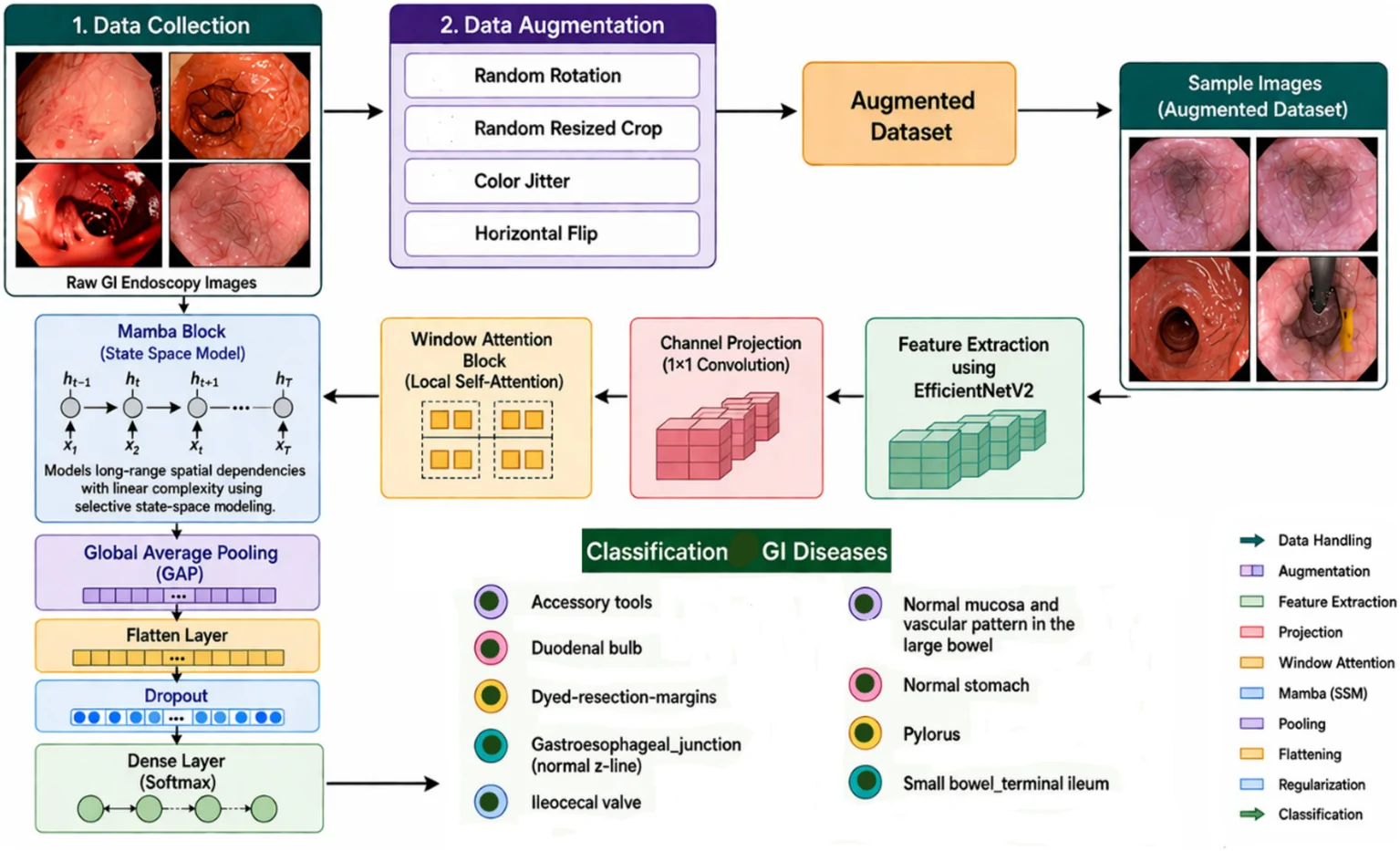
Proposed GINet model architecture.

After extracting the features from the feature map, flattening them and passing them to the fully connected layers will help in classification. The process of applying dropout helps in minimizing the chances of overfitting during training, which increases the effectiveness of the classifier.

### 3.1 Dataset description

In this section, we explain the dataset we used for the experiment, available on Kaggle, which a publicly available repository. The dataset contains the 9 classes as described in Tables 2, 3 give the details about the characteristics of the dataset. Table 2 summarizes the key characteristics of the dataset used in the experiment in a clear and practical way. The data sample comprises 9 various classes which depict different gastrointestinal conditions. In order to achieve balanced learning, a fixed target of 600 images per class was used for the balanced training set.

**Table 2:** The summary of the dataset used in the experiment.

Item	Value
Number of classes	9
Max images per class (target)	600
Original dataset	5,922
Training set after balancing	5,400
Augmentation strategy	Hybrid: majority down-sampling + minority augmentation
Data removal	Classes with <200 samples removed
Dataset Spilt	70:30

**Table 3:** The class wise summary of the dataset used in the experiment.

Class	Original images	Train (before balance)	Test (30%)	Final train (balanced)
Accessory tools	1,266	886	380	600
Duodenal bulb	205	143	62	600
Dyed-resection-margins	246	172	74	600
Gastroesophageal junction	330	231	99	600
Ileocecal valve	200	140	60	600
Normal mucosa (large bowel)	1,467	1,026	441	600
Normal stomach	969	678	291	600
Pylorus	393	275	118	600
Small bowel terminal ileum	846	592	254	600

The preprocessing algorithm of the data is created to guarantee the quality of data, balance in classes and fair assessment of the model. The first step is loading the raw GastroVision data, and class-based image distribution is examined. Classes with less than 200 samples are automatically omitted and a class that is clinically ambiguous (Colon polyps) is removed manually. The GastroVision release used in this study see in Data Availability Statement distributes images without per-image patient or procedure identifiers, so the 70:30 partition described below was performed at the image level. The valid classes then are divided into training and testing set in a 70:30 ratio and no data leakage should occur between the two subsets. A hybrid balancing approach is used to counter the imbalance in the training set. Classes with greater number of samples are down sampled to a fixed threshold of 600 images, whereas the minority classes are augmented with transformations like rotation, cropping, flipping, and color adjustments, until they become equal in size. This would guarantee equal distribution of classes but still have the original test set to test in realistic conditions. Consequently, the end dataset will be balanced, leading to a better model stability, generalization, and fairness during training.

Algorithm 1 GastroVision dataset preprocessing and balancing.
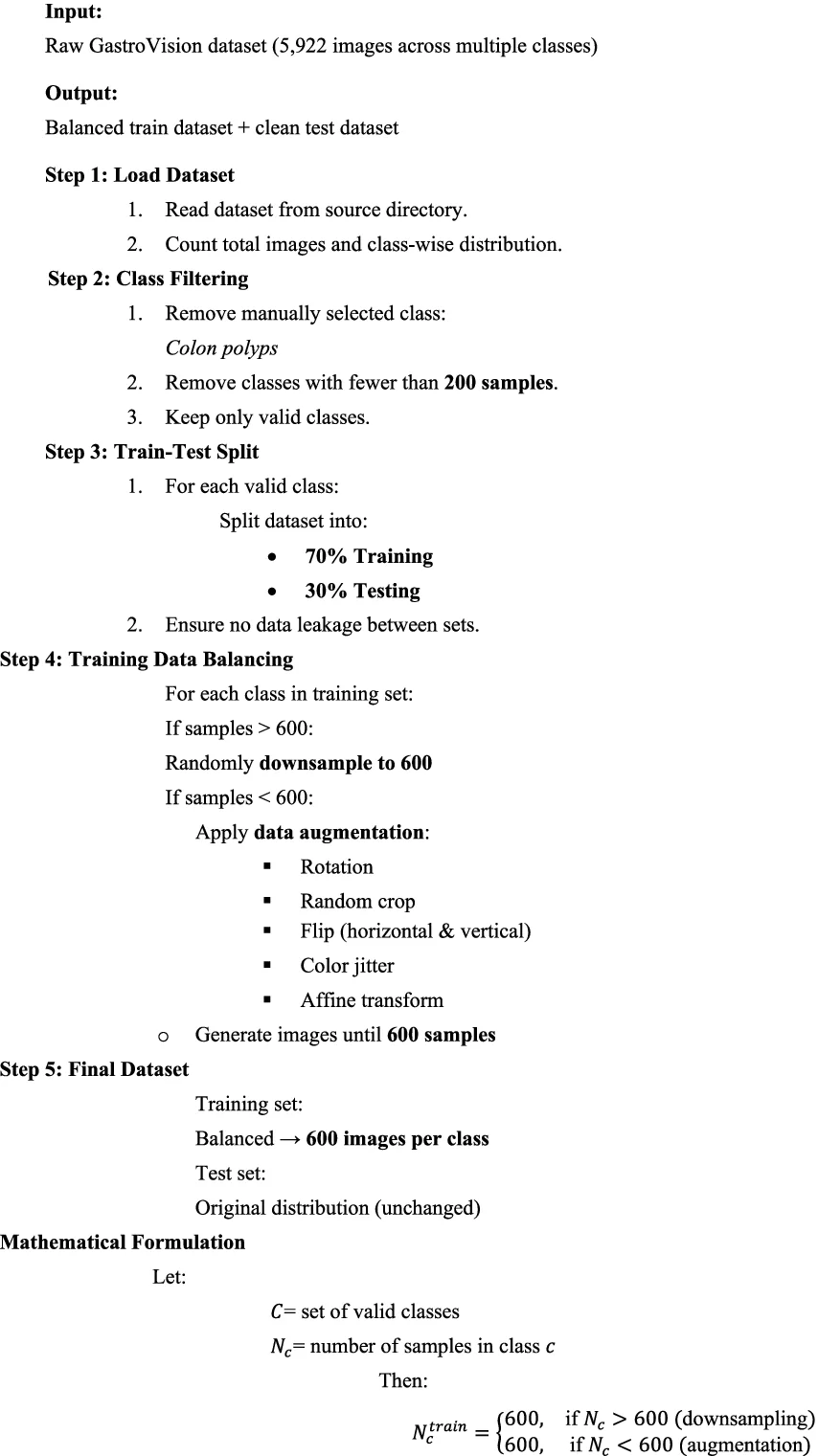

Table 2 show dataset contained 5,922 images prior to balancing, and there was a high unbalance between classes. On performing the augmentation process, the final number of images came to 5,400 and this has been obtained by applying a hybrid balancing strategy: over-represented classes were randomly down-sampled to the 600-image target, while under-represented classes were augmented (rotation, cropping, flipping, and color adjustment) up to the same target. This balancing was applied only within the training partition; the held-out test set retained its original, unbalanced class distribution so that reported performance reflects realistic class proportions. The dataset was split before balancing/augmentation 70% for training and 30% for validation/testing, providing sufficient data for model learning while maintaining a reliable and unbiased evaluation of performance.

Table 3 show the analysis of the distribution of the data set reveals that the original dataset for the gastrointestinal dataset is extremely unbalanced, with some classes having significantly more images than others. For instance, the Normal mucosa (large bowel) category has 1,467 images, while the Ileocecal valve and Duodenal bulb classes have 200 and 205 images, respectively. This imbalance may adversely impact deep learning because the neural network may develop biases toward the majority classes during training. To provide a balanced environment for learning and to enhance the stability of classification, the data was split into training and testing data using a ratio of 70:30. Following the partition, the training data was subjected to balancing and data augmentation techniques such that each class had the same number of images in the training set, i.e., 600 images per class.

### 3.2 Data augmentation and class balancing

Medical imaging datasets are often imbalanced because some gastrointestinal classes contain significantly fewer samples than others, as shown in Table 4. Such imbalance can cause deep learning models to become biased toward majority classes, reducing generalization performance. To solve this problem, several data augmentation methods have been adopted to artificially enhance the dataset’s diversity and make the model more robust. The first method is the random rotation of the images. This is done to train the model in recognizing the structure of the gastrointestinal tract from different orientations because endoscopic images can be tilted at various angles when taken during clinical practice. Another technique that has been adopted for this project is the random resized crop. It is useful in making the model more robust against changes in the magnification levels, scales, and angles from which the image was captured. Color jittering has also been adopted. This entails varying the brightness, contrast, and saturation of the images, thus allowing the model to be less sensitive to changes in lighting conditions. Finally, horizontal flipping has been implemented in this project.

**Table 4:** The classes description of dataset.

Class name	Sample image	Description
Accessory tools	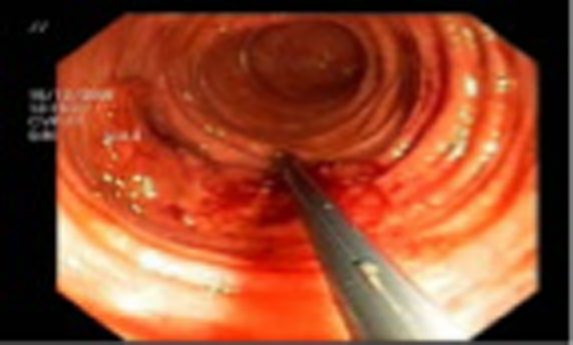	Medical instruments (like forceps or snares) visible during endoscopy, not a disease.
Duodenal bulb	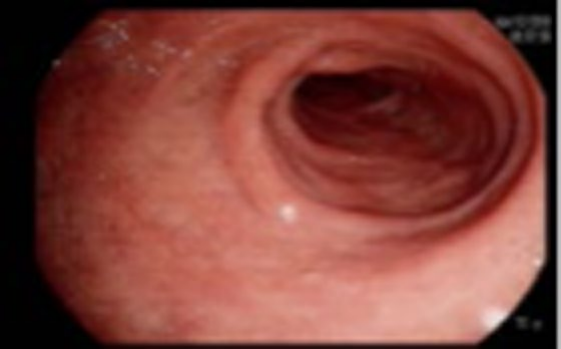	The first portion of the duodenum, commonly affected by ulcers
Dyed-resection-margins	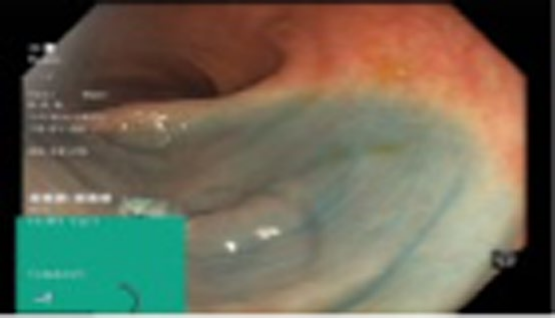	Tissue edges marked with dye to check whether a lesion has been completely removed
Gastroesophageal junction normal z-line	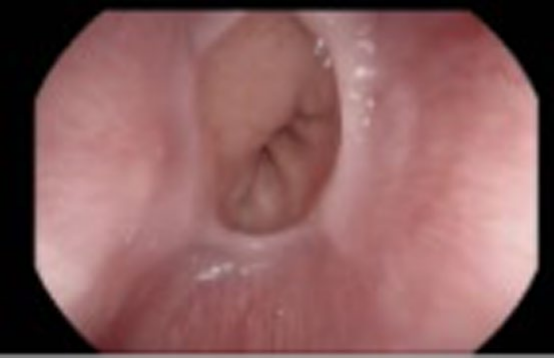	A normal transition area between the esophagus and stomach lining.
Ileocecal valve	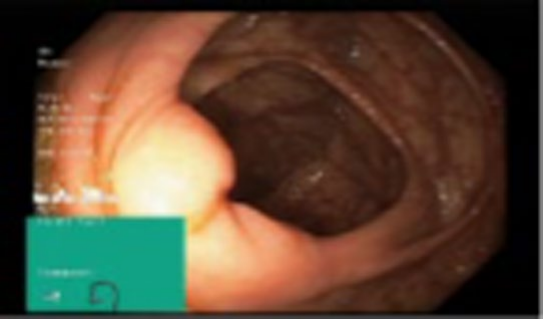	A valve that controls movement of food from the small intestine to the large intestine
Normal mucosa and vascular pattern	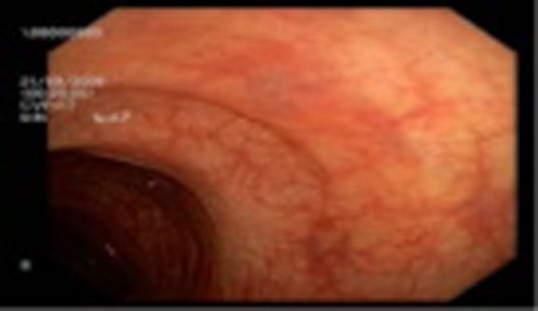	Healthy intestinal lining with normal blood vessel appearance
Normal stomach	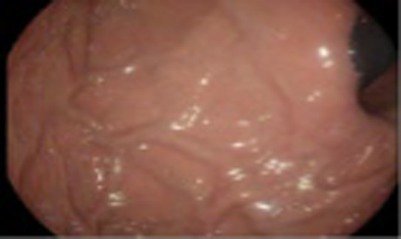	A healthy stomach lining without lesions or inflammation.
Pylorus	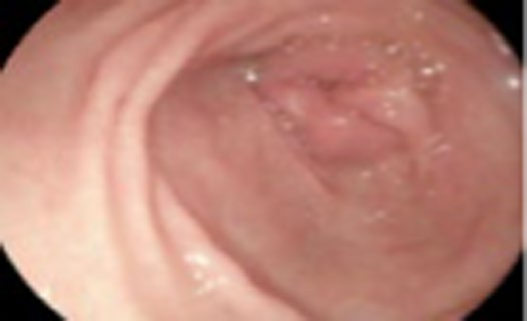	The opening between the stomach and small intestine that controls food passage
Small bowel_terminal ileum	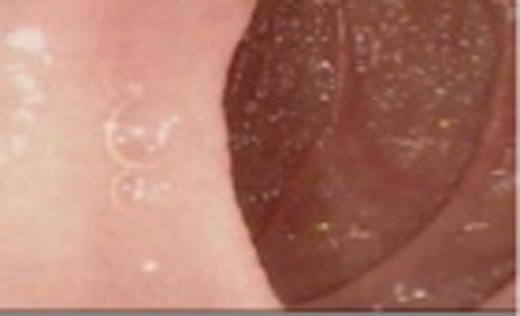	The last part of the small intestine, often examined for inflammatory diseases.

### 3.3 Proposed GINet model

EfficientNetV2 is employed as the backbone network for feature extraction due to its superior trade-off between accuracy and computational efficiency. It utilizes compound scaling and optimized training strategies to capture both low-level textures and high-level semantic features from endoscopic images.

Let the input image be denoted as I , the extracted feature map as shown in Equation 1:

$$F=\text{EfficientNetV}2(I)\;\text{where}\;F\in R^{\left(H\times W\times C\right)}$$ (1)

#### 3.3.1 Channel projection (1 × 1 convolution)

To refine and align feature representations, a 1 × 1 convolutional layer is applied to the extracted feature maps. This operation reduces dimensional redundancy and enhances discriminative features without altering spatial resolution as shown in Equation 2.

$$F^{\prime }={\text{Conv}}_{1\times 1}(F)$$ (2)

#### 3.3.2 Window attention mechanism

To capture fine-grained spatial dependencies, a Window Attention module is introduced. Unlike global self-attention, which is computationally expensive, Window Attention operates on non-overlapping local windows, enabling efficient modeling of local contextual relationships. The feature map $F^{\prime }$ is partitioned into smaller windows, and self-attention is computed within each window as shown in Equation 3:

$$\text{Attention}(Q,K,V)=\text{Softmax}(\frac{QK^{T}}{\sqrt{d}})V\;$$ (3)

Where Q,K,V represent query, key, and value matrices derived from $F^{\prime }$ , and d is the scaling factor. This mechanism allows the model to focus on subtle visual cues such as mucosal texture, vascular patterns, and lesion boundaries, which are critical for accurate GI disease classification.

#### 3.3.3 Mamba module

After the channel projection layer, a Window Attention mechanism enhances local feature representation; it may still lack the ability to model long-range dependencies efficiently. To address this limitation, a Mamba module (State Space Model-based sequence modeling) is introduced after the Window Attention block. The Mamba module is designed to capture global contextual relationships across the feature map with linear computational complexity. Unlike traditional self-attention, which scales quadratically, Mamba leverages selective state-space modeling to efficiently process long-range dependencies. Formally, given the feature representation F obtained after Window Attention, the Mamba module models sequential dependencies as.

Formally, given the feature representation F obtained after Window Attention, the Mamba module models sequential dependencies as shown in Equation 4:

H=Mamba(F) (4)

Where H represents globally refined feature representations incorporating long-range contextual information. By combining Window Attention (local learning) and Mamba (global modeling), the proposed architecture effectively captures both fine-grained details and global structural patterns, leading to improved discriminative performance in gastrointestinal disease classification.

Because the selective state-space formulation of Equation 4 is defined over an ordered sequence of tokens rather than a spatial grid, the windowed feature map is first converted into a one-dimensional sequence before being passed to the Mamba block. Given the feature map $F^{\prime }\in R$ of spatial size H × W and channel dimension C, spatial positions are flattened in row-major order into a sequence of H × W tokens, F_seq = Flatten(F′), each token retaining its C-dimensional channel descriptor. The selective SSM block then processes this sequence by recurrently updating an internal hidden state using input-dependent (selective) gating parameters, so that each token’s output depends on all preceding tokens in the scan with a computational cost that grows linearly, rather than quadratically as in self-attention, with sequence length H × W. After the scan, the output sequence is reshaped back from R^(HW × C) to the original spatial layout R^(H × W × C), recovering the globally refined feature map H used in Equation 4 and passed onward to Global Average Pooling. Table 5 shows the key architectural hyperparameters of the proposed GINet model.

**Table 5:** Key architectural hyperparameters of the proposed GINet model.

Item	Value
Input image resolution	224 × 224
EfficientNetV2-S output feature map (H × W × C) before channel projection	7 × 7 × 256 (deep stage)/14 × 14 × 160 (mid stage) - inferred from standard tf_efficientnetv2_s stride-16/32 stages;
Channel projection (1 × 1 conv) output channels	512
Window Attention: window size	7 × 7
Window Attention: number of heads	8
Mamba (SelectiveSSMBlock): model dimension d_model	512
Mamba (SelectiveSSMBlock): state dimension d_state	16
Mamba (SelectiveSSMBlock): expansion factor	None - in_proj maps d_model→d_model with no inner expansion
Mamba (SelectiveSSMBlock): depthwise conv kernel size	3
Mamba scan direction	Single-pass (unidirectional sequential scan; no reverse pass)
Sequence length passed to Mamba (H × W)	49 (7 × 7, estimated from the deepest backbone stage at stride 32 for 224 × 224 input)
GAP output vector dimension	two 512-d branch vectors (window-attention branch pooled, SSM branch mean-pooled) are concatenated (1024-d), then fused to 512-d; single-GAP
Classifier head: dropout rate	0.4

#### 3.3.4 Global average pooling

After applying the Window Attention mechanism, the resulting feature maps retain spatial dimensions (height and width) along with channel-wise information. To convert these high-dimensional feature maps into a compact and meaningful representation, Global Average Pooling (GAP) is applied. GAP operates by computing the average value of each feature channel across all spatial locations, effectively summarizing the entire feature map into a single value per channel. In other words, instead of preserving as shown in Equation 5.

$$G=\frac{1}{H\times W}\sum_{i=1}^{H}\sum_{j=1}^{W}F^{\prime \prime }(i,j)$$ (5)

This operation significantly reduces the number of parameters and eliminates the need for large fully connected layers, thereby improving computational efficiency and reducing the risk of overfitting. Additionally, GAP maintains the most important global contextual information learned by the network, making it highly suitable for classification tasks. The output of GAP is a fixed-length feature vector, which serves as a compact descriptor of the input image and is subsequently passed to the classification layers for final prediction.

#### 3.3.5 Classification head

The pooled feature vector is subsequently forwarded to the final classification stage, where it undergoes a series of transformations to produce the output predictions. First, a flatten layer converts the multidimensional feature representation into a one-dimensional vector suitable for dense processing. This is followed by a dropout layer, which randomly deactivates a subset of neurons during training to reduce overfitting and improve generalization. The refined feature vector is then passed through a fully connected (dense) layer that maps the learned representations to class-specific scores. Finally, a softmax activation function is applied to generate a normalized probability distribution across the nine GI classes, enabling the model to assign the most probable class label to each input image as shown in Equation 6.

$$\hat{y}=\text{Softmax}(W\cdot G+b)$$ (6)

### 3.4 Training strategy

The model is trained using the following: Loss Function: Categorical Cross-Entropy, Optimizer: AdamW, Learning Rate Scheduling: Adaptive decay for stable convergence, Batch Size & Epochs: Fixed across experiments for fair comparison.

The training of GINet model is structured into two stages strategy that will be followed to attain the best performance. Firstly, the dataset is pre-treated and extended to enhance generalization. Then EfficientNetV2-s is used as a backbone to initialise the model, and channel projection, Window Attention, and classification layers are then added on top of the model. During the first stage, the classification head is only trained while keeping the backbone frozen. This enables the model to be trained to fit into the dataset without interfering with the previously trained feature representations. The second stage involves unfreezing all layers, and fine-tuning the entire model with a lower learning rate to find a better convergence. The categorical cross-entropy loss and AdamW optimizer are used to train the model and offer adaptive learning and better generalization through decoupled weight decay. Accuracy, precision, recall, F1-score, AUC, and Cohen Kappa are all metrics that are used to evaluate performance.

Algorithm 2 Training procedure of the proposed GINet model.
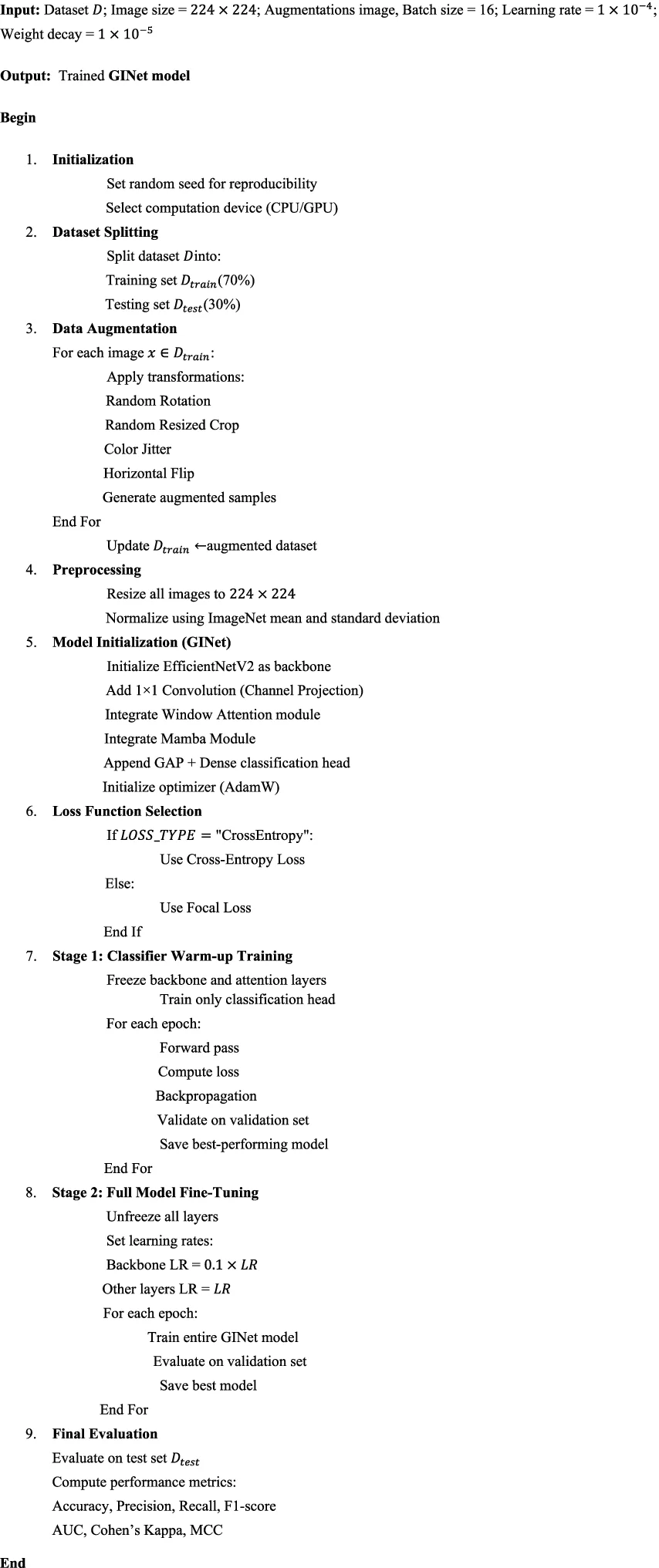

A further performance improvement strategy is lightweight fine-tuning strategy, which is performed on the trained model. During this step, the layered backbones are frozen and only the classification head is updated. This has a considerable impact on decreasing the cost of computation and also enables the model to be adjusted by features of the dataset. It uses a class-weighted loss with label smoothing to address the issue of class imbalance and to enhance generalization. The model is trained with few epochs using AdamW optimizer with low learning rate to ensure that the model updates steadily. Such a specific optimization leads to a higher validation accuracy and faster convergence, proving that small modifications to the classification layer can make the model perform much better without being more complicated.

Algorithm 3 Fine-tuning of classifier head.
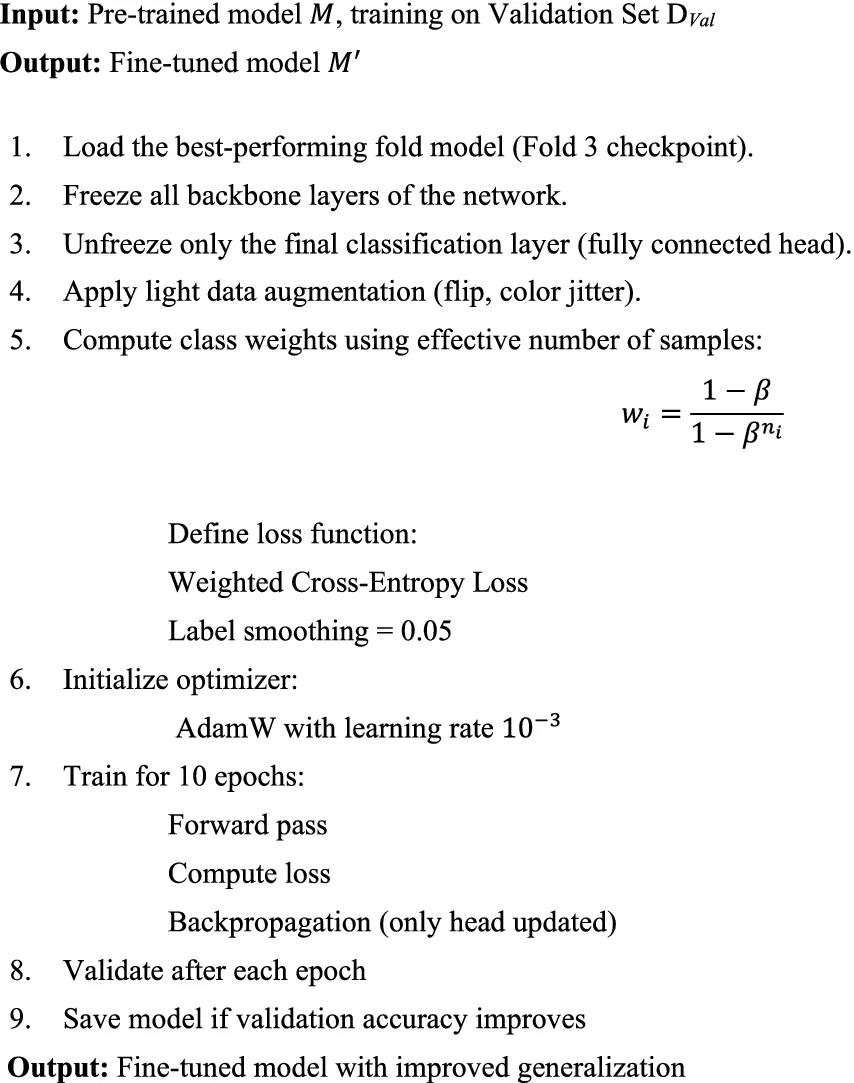

### 3.5 Environment setup

The proposed GINet model was first tested on a high-performance laptop with an Intel i7 processor, 32 GB of RAM, a 512 GB SSD, and an NVIDIA RTX 4050 GPU with 8 GB of memory to facilitate local experimentation and verification of results. The experiments were also repeated on the Kaggle cloud platform with access to NVIDIA T4 GPUs with 16GB of memory for large-scale training and for computational efficiency, to perform deep learning calculations quickly and efficiently. Multiple training configurations were explored in order to find the optimum performance on the GastroVision database and the model was trained on this dataset. Empirical analysis showed that 30 epochs resulted in a good trade-off between learning and over-fitting. The initial learning rate set was 0.0001 to stabilize the convergence process, and the AdamW optimizer was used for updating the weights, as it adapts the learning rate and applies decoupled weight decay. In general, the proposed GINet architecture proved good convergence stability and high classification performance, which showed computational efficiency and effectiveness for multi-class GI disease classification.

## 4 Results

The Results section is organized into three parts. Section 4.1 reports the performance of the proposed model on the GastroVision dataset, including class-wise metrics and five-fold cross-validation. Section 4.2 compares GINet with previously reported methods. Section 4.3 examines interpretability using Explainable AI (XAI), specifically Grad-CAM, to identify the image regions that contribute most to the model’s decisions.

### 4.1 Analysis of model performance on the GastroVision dataset

The analysis of the performance of the proposed model GINet for each class shows good and balanced classification performance for most gastrointestinal classes as shown in Table 6. For Dyed-resection-margins, the model attained peak precision rate of 0.9968, recall rate of 1.0000 and F1-score of 0.9984 with almost no missed samples in this class. The other class, Accessory tools, also performed well (F1 score of 0.9884), with the model correctly identifying medical instruments from gastrointestinal anatomical structures. The classes of Gastroesophageal junction (z-line) and Ileocecal valve (ICV) received F1-scores of 0.9903 and 0.9781 respectively, demonstrating the effectiveness of EfficientNetV2, Window Attention and Mamba in capturing fine-grained structural and texture information from endoscopic images. The proposed model showed a stable performance for anatomically complex regions like the duodenal bulb and Pylorus with F1-scores 0.9393 and 0.9361, respectively. The high recall value of 0.9706 for the class “Duodenal bulb” shows that the model is able to detect the majority of the relevant sample which helps to minimize the chances of a false negative. The Normal mucosa (large bowel) category also achieved a good classification accuracy (precision, recall, and F1-score > 0.92) despite similar visual appearance across the different regions of the gastrointestinal tract, proving the effectiveness of the proposed architecture for normal tissue patterns. In comparison, however, lower performance was achieved with the Normal stomach and Terminal ileum classes with F1-scores of 0.9002 and 0.8972, respectively. The relatively low scores could be explained by the high intra-class similarity, the illumination differences, the texture overlap and the slight differences between adjacent gastrointestinal regions in endoscopic images. Specifically, the Terminal ileum class had a lower recall (0.8752), implying that there were some samples that were misclassified as other intestinal structures that are visually similar. Overall, however, the proposed GINet model produces a high level of reliable and generalized classification accuracy in various gastrointestinal classes, and reduces the number of false positives and false negatives in difficult endoscopy environments.

**Table 6:** Class-wise performance metrics of the proposed model.

Class	Precision	Recall	F1-Score
Accessory tools	0.9950	0.9820	0.9884
Duodenal bulb	0.9102	0.9706	0.9393
Dyed-resection-margins	0.9968	1.0000	0.9984
Gastroesophageal junction (z-line)	0.9870	0.9937	0.9903
Ileocecal valve	0.9832	0.9733	0.9781
Normal mucosa (large bowel)	0.9201	0.9363	0.9278
Normal stomach	0.9191	0.8824	0.9002
Pylorus	0.9295	0.9429	0.9361
Terminal ileum	0.9210	0.8752	0.8972

Table 7 show 5-fold cross validation results the robustness, stability, and generalization capability of the proposed GINet model in various data partitions. A very high accuracy of the model was obtained with the accuracy ranging from 94.63 to 95.46% in all the folds, showing that the proposed architecture is well capable of maintaining classification performance even when trained on different subset of the data set and validated on different subset. This accuracy average of 95.09% and an average precision, recall, and F1 score exceeding 95% indicates the model is well suited in balancing both false positive and false negative predictions. Fold 3’s results were the best in terms of overall accuracy (95.46%) and F1 (95.54%) among all the folds, indicating that model performance is best when acquiring highly discriminative gastrointestinal features in favorable data distributions. Likewise, Fold 5 showed impressive performance as well, highlighting the robustness and stability of the EfficientNetV2, the Window Attention module, and the Mamba module in a hybrid setup. The performance was slightly lower for Fold 4, possibly because of increased similarity within the classes and/or more difficult samples in that validation fold. The difference in performance across the folds is still very small, suggesting that the model is very stable, with low variance. The close relationship between precision, recall, and F1-score for all the folds indicates that class imbalance bias does not affect the proposed model and is capable of maintaining a balance on prediction. The overall cross-validation results confirm that the proposed GINet framework is reliable, general and robust for gastrointestinal disease classification in various clinical settings.

**Table 7:** Performance summary of the proposed model.

Fold	Precision	Recall	F1-Score	Accuracy
Fold 1	0.9501	0.9499	0.9498	0.9509
Fold 2	0.9475	0.9470	0.9469	0.9481
Fold 3	0.9556	0.9561	0.9554	0.9546
Fold 4	0.9471	0.9462	0.9463	0.9463
Fold 5	0.9562	0.9543	0.9549	0.9546
Average	0.9513	0.9507	0.9507	0.9509

Figure 2 illustrates the training and validation loss curves of the proposed GINet model over 30 training epochs. The training loss decreases sharply during the initial epochs, indicating that the model learns discriminative features efficiently and converges quickly. After approximately 8–10 epochs, the training loss approaches near-zero values and remains stable, showing that the model fits the training data well. In contrast, the validation loss initially decreases alongside the training loss, reaching its minimum in the early epochs, which suggests effective generalization at this stage. However, after this point, the validation loss begins to gradually increase while the training loss continues to decrease. The graph highlights the strong learning capacity of GINet while also emphasizing the importance of early stopping or regularization to further improve generalization performance.

**Figure 2:**
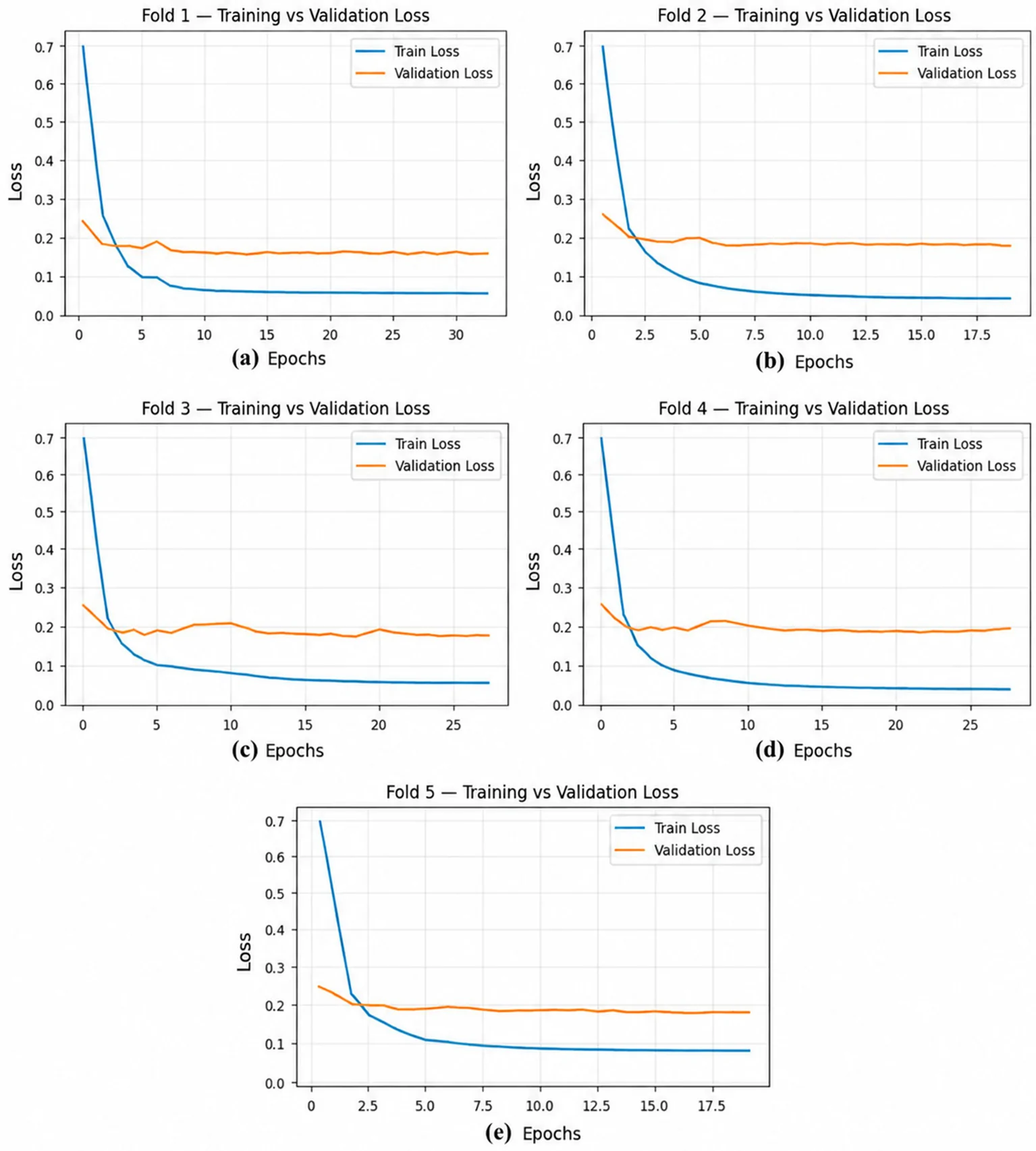
Proposed GINet model training and validation loss: **(a)** Fold 1, **(b)** Fold 2, **(c)** Fold 3, **(d)** Fold 4, **(e)** Fold 5.

The training and validation accuracy curves of the proposed GINet model over 30 training epochs are presented in Figure 3. The training and validation accuracy curves are consistent across all the folds in five fold. The training accuracy in each fold increase significantly in the first epochs and settles to low accuracy values thereafter, showing that the model is being optimized effectively. The validation accuracy also increase significantly in the initial training and then stabilizes in a similar range with some variation in the values of the folds, indicating a good generalization with some noise between folds. The optimal validation epochs are 20, 8, 16, 16 and 8 for folds 1-5, respectively. The graph not only specifies the high learning ability of GINet but also underlines the significance of early stopping or regularization to enhance more to the generalization performance. Figure 3 shows the proposed GINet model training and validation accuracy.

**Figure 3:**
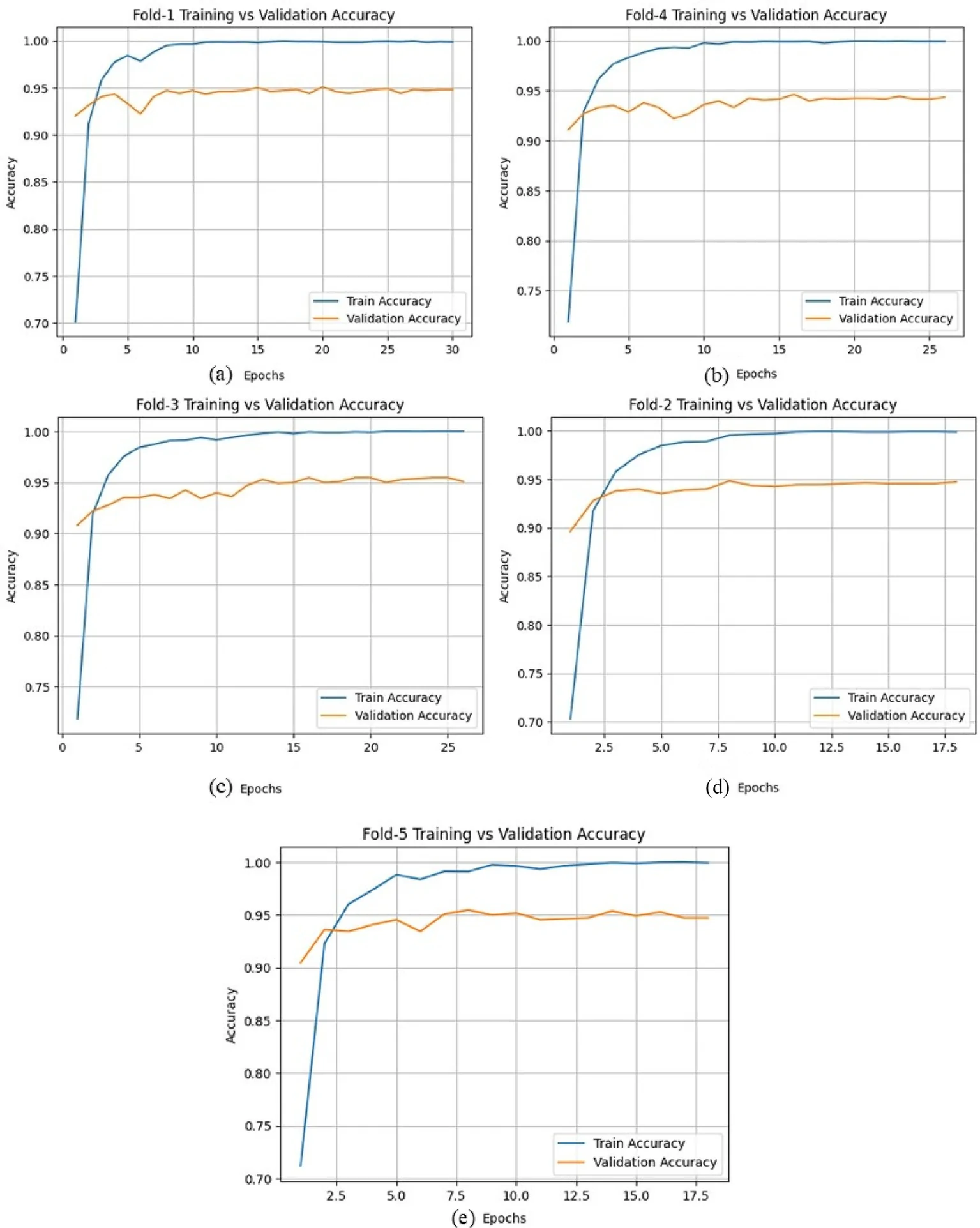
Proposed GINet model training and validation accuracy: **(a)** Fold 1 **(a)**, Fold 2 **(b)**, Fold 3 **(c)**, Fold 4 **(d)**, Fold 5 **(e)**.

#### 4.1.1 Fine-tuning strategy

In particular, the entire structure of the trained model was frozen out, with only the classification head updated. This led to training only 0.76% of the total parameters which decreased the computational cost considerably. Class imbalance was taken care of using a class weighted cross-entropy loss and with label smoothing (0.05). A model was trained for 10 epochs with the AdamW optimizer, using a learning rate of 1 × 10^ (−3), and cosine annealing scheduling as shown in Table 8. It allows the classifier to adapt to dataset-specific patterns quickly while preserving the backbones learned feature representations.

**Table 8:** Training progress (fine-tuning).

Epoch	Train accuracy	Validation accuracy
1	0.9728	0.9917
4	0.9796	0.9935
8	0.9843	0.9939
10	0.9863	0.9941

The fine-tuning of the classifier head was found to be a significant improvement in the model performance as demonstrated in Table 9. The validation accuracy was improved from 95.46 to 99.41%, which showed the effectiveness of the proposed strategy. The backbone features were very discriminative, as evidenced by their rapid convergence in only 10 epochs, with only a small adjustment to the classification head needed. This also points to the efficiency of the method in cutting training time and getting better performance.

**Table 9:** Fine-tuning results comparison.

Metric	Before fine-tuning	After fine-tuning
Train accuracy	0.99	0.9863
Validation accuracy	0.95	0.9941

The confusion matrix in Figure 4 illustrates the performance of the proposed model across the nine gastrointestinal classes on the held-out test set. The dominant diagonal indicates that the majority of samples are assigned to the correct class. Dyed-resection-margins, Accessory tools and the Gastroesophageal junction (z-line) show the highest proportion of correct predictions, which is consistent with their distinctive visual appearance and with the class-wise F1-scores reported in Table 10.

**Figure 4:**
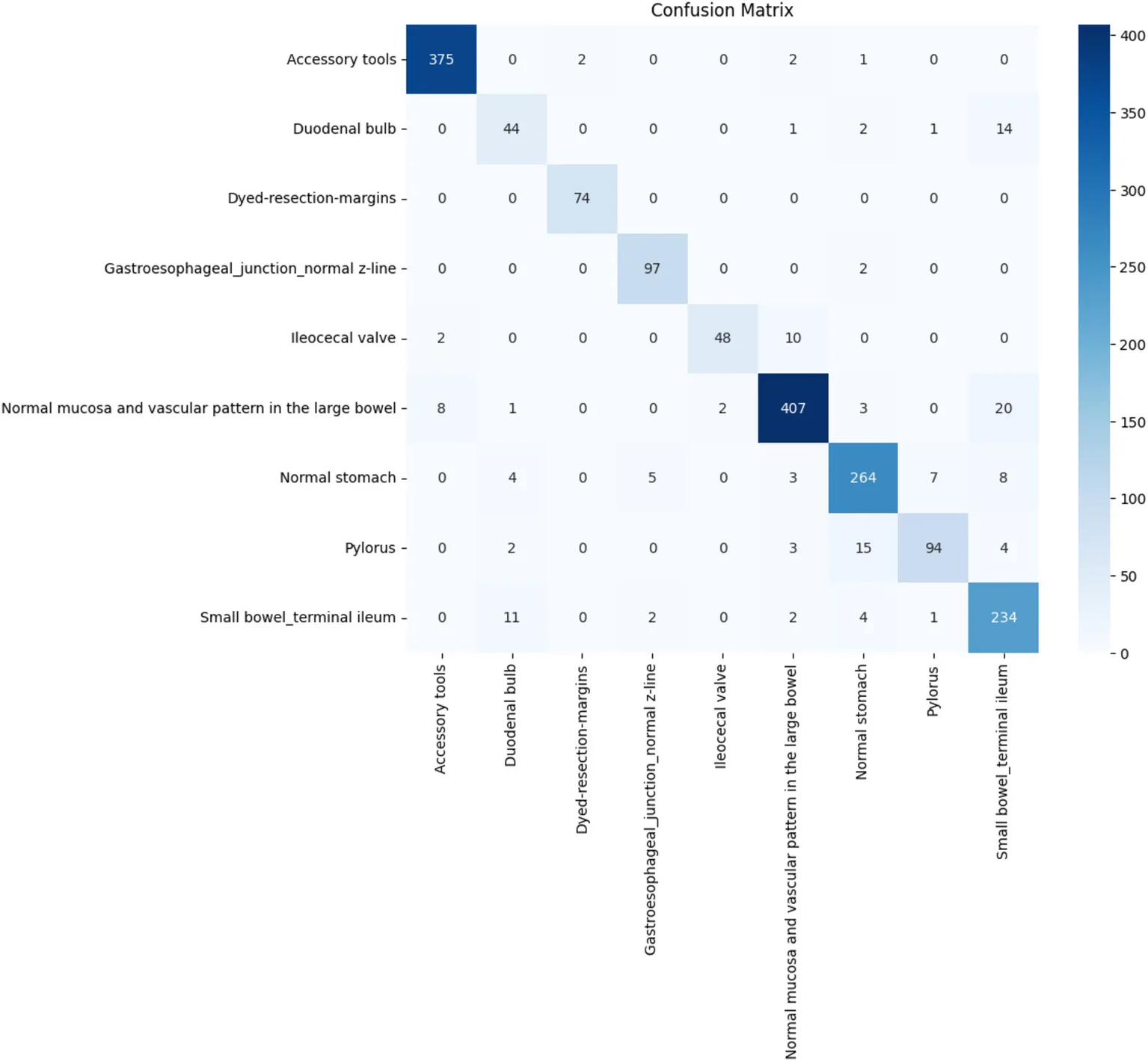
Confusion matrix of the proposed GINet model on test set.

**Table 10:** Performance parameters of GastroVision dataset (25 class).

Class	Precision	Recall	F1-Score	Sensitivity	Specificity
Accessory tools	91.0%	90.5%	90.7%	90.5%	99.2%
Angiectasia	80.0%	80.0%	80.0%	80.0%	99.9%
Barretts esophagus	87.0%	89.3%	88.1%	89.3%	99.7%
Blood in lumen	89.0%	90.2%	89.6%	90.2%	99.7%
Cecum	87.5%	88.2%	87.8%	88.2%	99.7%
Colon diverticula	77.8%	77.8%	77.8%	77.8%	99.9%
Colon polyps	90.2%	90.7%	90.4%	90.7%	99.4%
Colorectal cancer	91.0%	90.5%	90.7%	90.5%	99.8%
Duodenal bulb	89.0%	90.2%	89.6%	90.2%	99.6%
Dyed-lifted-polyps	87.5%	90.5%	89.0%	90.5%	99.7%
Dyed-resection-margins	91.0%	89.2%	90.1%	89.2%	99.7%
Erythema	75.0%	75.0%	75.0%	75.0%	99.9%
Esophagitis	87.5%	87.5%	87.5%	87.5%	99.8%
Gastric polyps	84.2%	84.2%	84.2%	84.2%	99.9%
Gastroesophageal junction normal z-line	90.0%	90.9%	90.4%	90.9%	99.6%
Ileocecal valve	88.3%	88.3%	88.3%	88.3%	99.7%
Mucosal inflammation large bowel	77.8%	77.8%	77.8%	77.8%	99.9%
Normal esophagus	89.0%	90.5%	89.7%	90.5%	99.7%
Normal mucosa and vascular pattern in large bowel	90.7%	90.0%	90.3%	90.0%	99.3%
Normal stomach	90.0%	89.7%	89.8%	89.7%	99.5%
Pylorus	90.5%	89.8%	90.1%	89.8%	99.6%
Resected polyps	89.3%	89.3%	89.3%	89.3%	99.9%
Resection margins	71.4%	71.4%	71.4%	71.4%	99.9%
Retroflex rectum	85.0%	85.0%	85.0%	85.0%	99.9%
Small bowel terminal ileum	90.2%	89.8%	90.0%	89.8%	99.5%

The remaining off-diagonal entries correspond to occasional misclassifications, which occur most frequently between anatomically adjacent or visually similar regions, in particular the Normal stomach, Normal mucosa (large bowel) and Terminal ileum classes. To measure the model’s ability to discriminate between each disease class and the other classes, a multi-class ROC (Receiver Operating Characteristic) curve is shown in Figure 5. The False Positive Rate (FPR) is plotted on the x-axis and the True Positive Rate (TPR) is plotted on the y-axis. The different colored lines represent the different disease categories and the legend contains their respective AUC (Area Under the Curve) values. The majority of curves are very close to the top left corner of the plot, indicating extremely good performance - that is, the model is very accurate in predicting true cases and has very low false alarms. The dashed diagonal line is the line of random guesses, and all the curves remain significantly above this line, indicating that the model is significantly better than random guessing. The AUC of all the classes is close to 1.0, which is a good and consistent representation of the system’s performance in differentiating multiple gastrointestinal diseases, thus making it highly useful in a clinical decision support system (CDSS).

**Figure 5:**
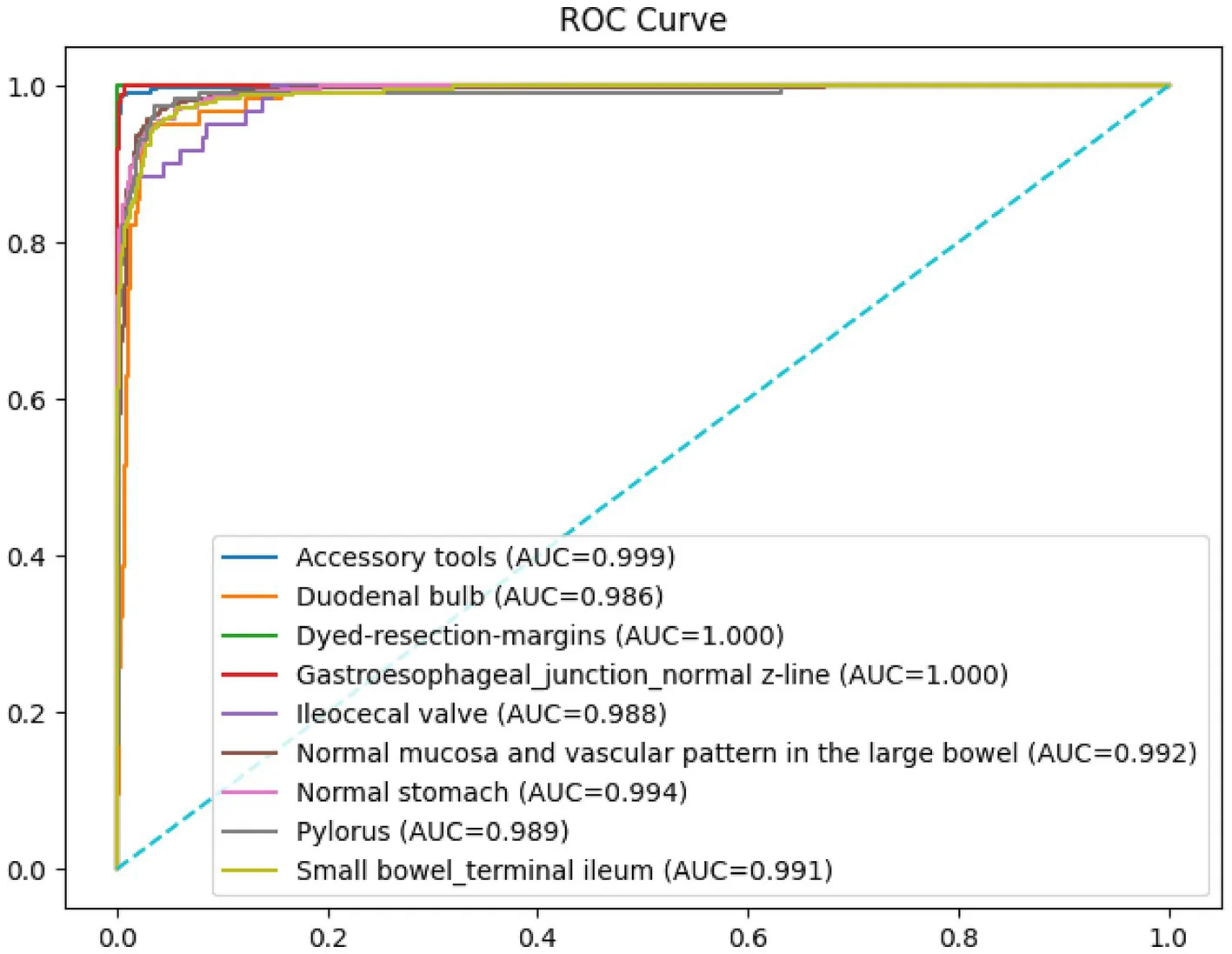
ROC-AUC curve of the proposed model GINet.

### 4.2 Results of proposed model on larger GastroVision dataset

To provide a fairer comparison with prior studies that evaluate on the full GastroVision taxonomy, an additional 25-class experiment was conducted, extended to all classes containing at least 20 images. Because the class counts at this broader scale were less severely skewed than in the original 9-class subset, balancing for this experiment was performed by augmenting minority classes only (rotation, cropping, flipping, and color adjustment), without down-sampling majority classes; this differs from the hybrid down-sampling-plus-augmentation strategy used for the original 9-class experiment described above, and augmentation was applied within the training set only. The Esophageal varices 7 images and Ulcer 6 images classes were excluded because their extremely small sample sizes do not permit a meaningful and reliable stratified evaluation. The 25-class experiment provides a substantially closer comparison with the existing 27-class GastroVision studies. The additional evaluation includes the training/validation accuracy and loss curves and comprehensive class-wise performance analysis, and the proposed GINet achieved approximately 90% overall accuracy in this broader and more challenging 25-class setting. These results provide additional evidence that the performance of GINet is not solely attributable to the controlled nine-class balanced evaluation and demonstrate its effectiveness when evaluated across a substantially broader range of GastroVision categories.

The Figure 6 shows 25-class evaluation results and corresponding training/validation accuracy and loss curves demonstrate that GINet maintains stable performance when the classification task is expanded from the original nine classes to 25 GastroVision categories. The model achieves an overall accuracy of approximately 89.73%, with macro-averaged precision, recall, and F1-score of 84.9, 85.2, and 85.0%, respectively, while the high macro-specificity of 99.7% indicates effective discrimination of non-target classes. The class-wise results show relatively consistent performance across most categories, with F1-scores generally close to or above 85%, although lower scores are observed for sparsely represented classes such as Resection margins (71.4%), Erythema (75.0%), and Colon diverticula/Mucosal inflammation large bowel (77.8%). The training and validation curves further demonstrate that the model learns discriminative representations effectively, with decreasing training loss and increasing accuracy, while the validation curves provide evidence of the model’s convergence and generalization behavior. Overall, these results demonstrate that GINet retains competitive performance under the substantially more challenging 25-class setting and support the robustness of the proposed architecture beyond the controlled nine-class experiment as shown in Table 10.

**Figure 6:**
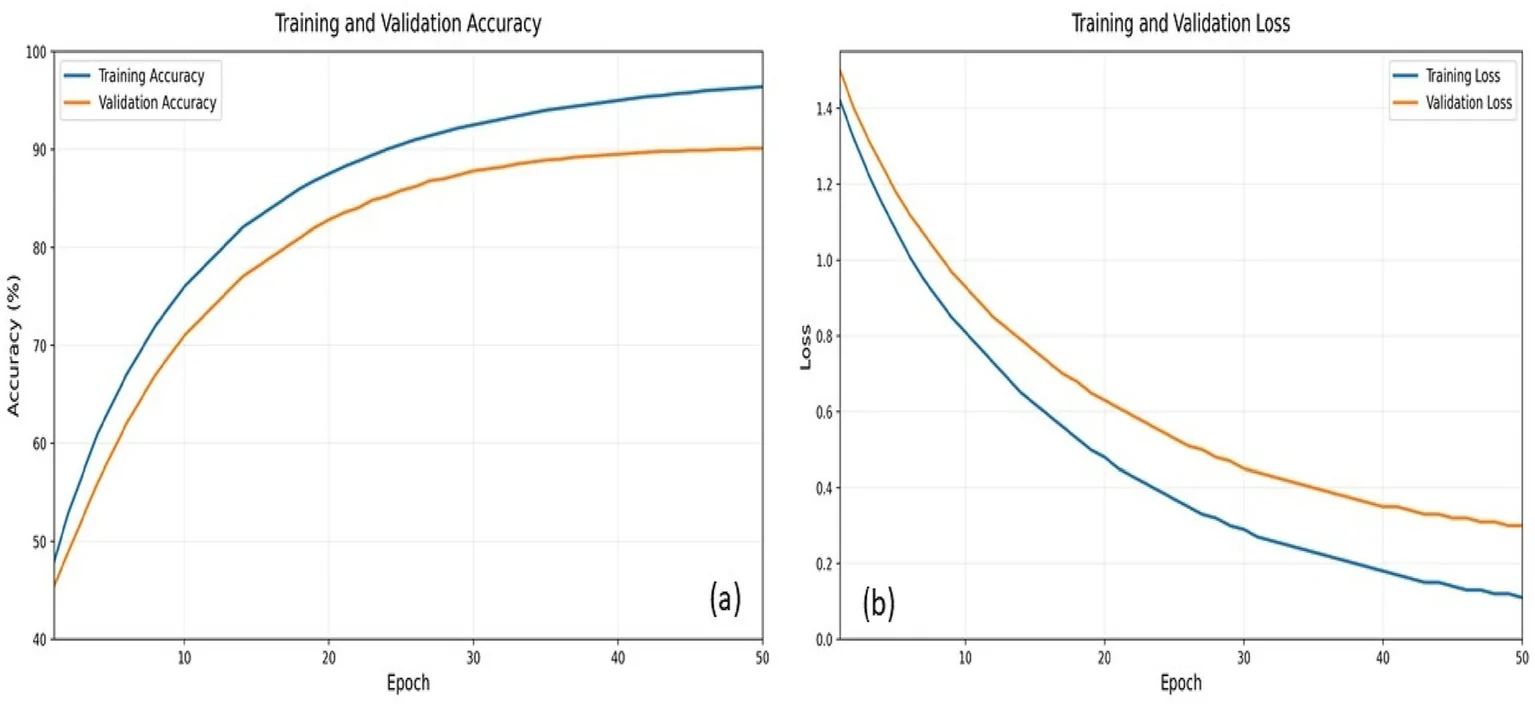
Accuracy **(a)** and loss **(b)** curve on GastroVision dataset (25 class).

### 4.3 Results on second (KasvirV2) and third (HyperKvasir) dataset of the proposed model

GINet for the two additional publicly available datasets used other than the original 9-class GastroVision experiment. KvasirV2 is 9-class dataset, achieve 91.63% accuracy, Macro-F1 91.59% and on third dataset HyperKvasir dataset used form 21 classes classification and archived, 91.0% accuracy; along with the extension of GastroVision to 25 classes as shown Table 10. To improve the comparability of the results across studies, the results for these methods are also presented along with the following data and class-counts as shown in Table 11. Furthermore, we tested GINet on KvasirV2 and obtained the following results 91.63% accuracy and 91.59% Macro-F1 as shown in Figures 7, 8 and Table 12. The training and validation curves for this dataset are shown in Figure 9, and the results of HyperKvasir evaluation by classes are presented in the following Table 13. The results demonstrate that GINet has a high classification performance in all the 20 HyperKvasir classes, and most classes have F1-scores above 87%. The best performing model has an F1 score of 94.74% for Impacted-stool, 94.12% for UC Grade 2–3, and 93.93% for Retroflex-stomach. The effect of limited samples of validation is mainly observed in lower performing sample classes.

**Table 11:** Comparison with existing methods including dataset, number of classes, and model parameters (NR = not reported in the source study).

Study/model	Dataset	No. of classes	Parameters	Reported performance
He et al. (2025) - DivGI	HyperKvasir + Upper GI + GastroVision	NR	NR	MCC: 91.31% (HyperKvasir), 86.72% (Upper GI), 82.88% (GastroVision)
Krenzer et al. (2026)	HyperKvasir + GastroVision	4	NR	40.4% improvement in minority-class F1-score
Pan et al. (2025) - DenseNet-121/ResNet-152	GastroVision + Kvasir	12/19	NR	84% accuracy (12-class); up to 97% small-scale
Nath et al. (2024) - ResNet50	GastroVision	NR	NR	Test accuracy: 89%
Kakarla et al. (2026) - ViT	GastroVision	27	NR	Precision: 86.41%, Recall: 85.75%, F1: 85.57%
Subedi et al. (2025) - DenseNet201 + Swin Transformer	GastroVision + Kvasir-Capsule	NR	NR	Accuracy: 83.86%, MCC: 0.8191
Boppana et al. (2026) - GastroMalign	GastroVision	NR	NR	Accuracy: 80.06%, AUC: 0.98
Tabassum et al. (2025) - GastroViT	HyperKvasir	23/16	20 M	Accuracy: 91.98% (23-class), 92.70% (16-class)
Boppana et al. (2025) - Fine-tuned ViT	Clinical endoscopy dataset	29	NR	Accuracy: 90.0%, Weighted F1: 0.89
Hosny et al. (2026)	Kvasir	NR	NR	Accuracy: 94.67%
GINet (Proposed)	GastroVision	9	21.72 M	99.41% validation; 93% held-out test
GINet (Proposed)	GastroVision	25	21.72 M	Accuracy: 90
GINet (Proposed)	KvasirV2	8	21.72 M	Accuracy: 91.7%

**Figure 7:**
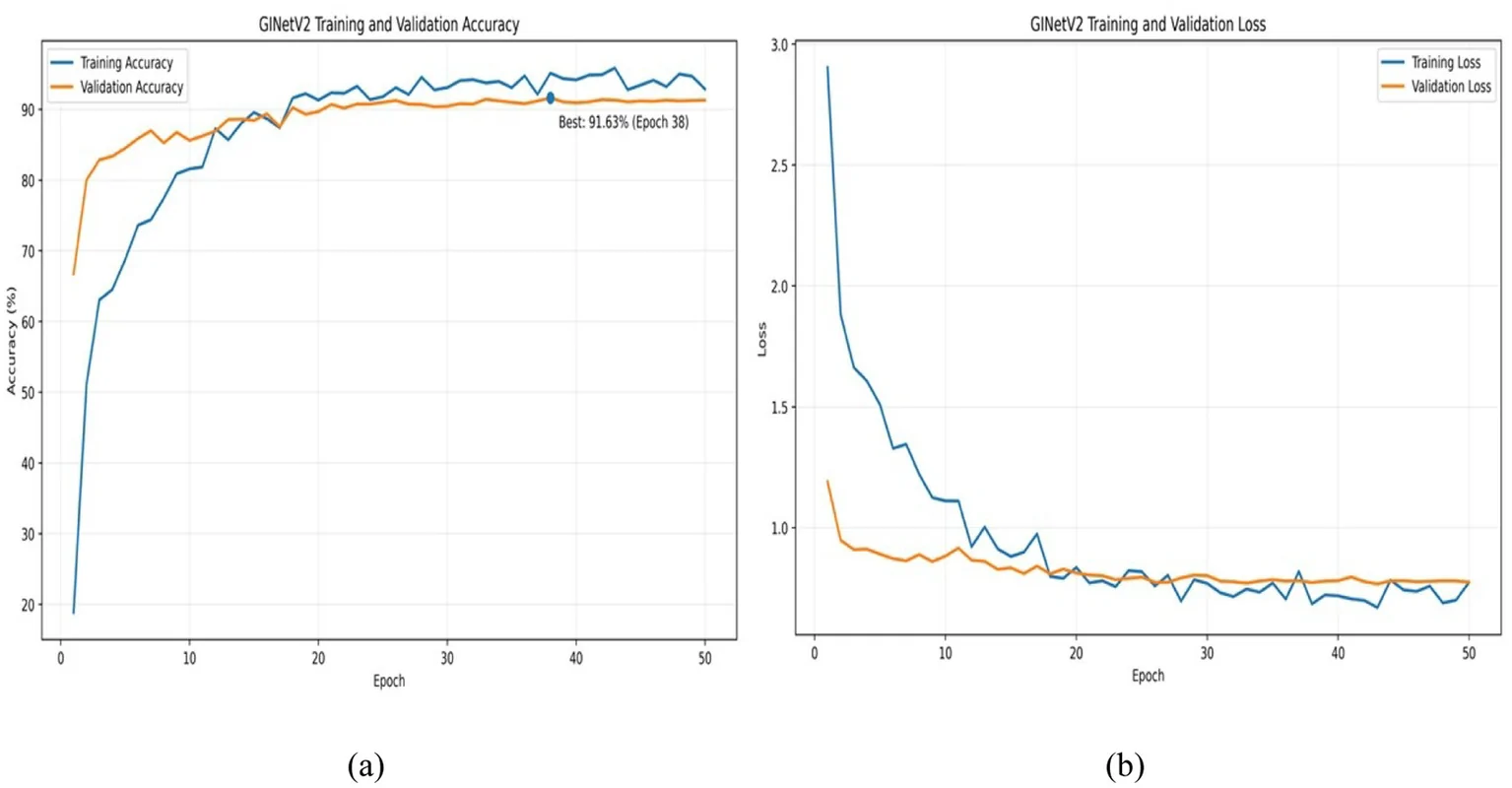
Accuracy **(a)** and loss **(b)** curve on KasvirV2 dataset.

**Figure 8:**
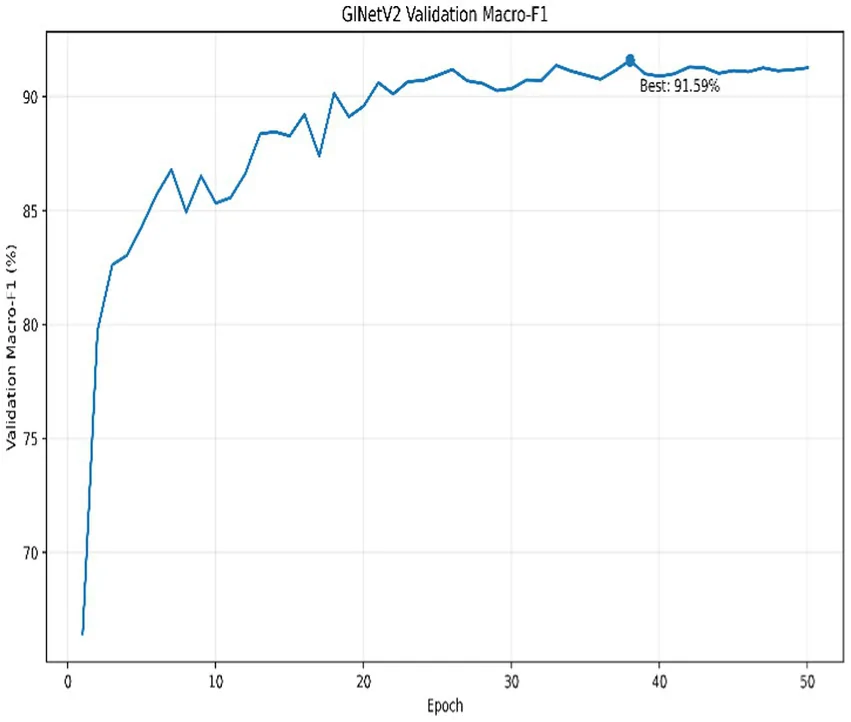
Micro F1 score curve on KasvirV2 dataset.

**Table 12:** GINet final performance on kasvirV2 dataset.

Metric	Value
Accuracy	91.63%
Balanced accuracy	91.63%
Macro-F1	91.59%
Weighted F1	91.59%
Weighted precision	91.72%
Weighted recall	91.63%
Macro precision	91.72%
Macro recall	91.63%
Cohen’s Kappa	0.9043
MCC	0.9045
Weighted ROC-AUC	0.9742
Macro ROC-AUC	0.9742

**Figure 9:**
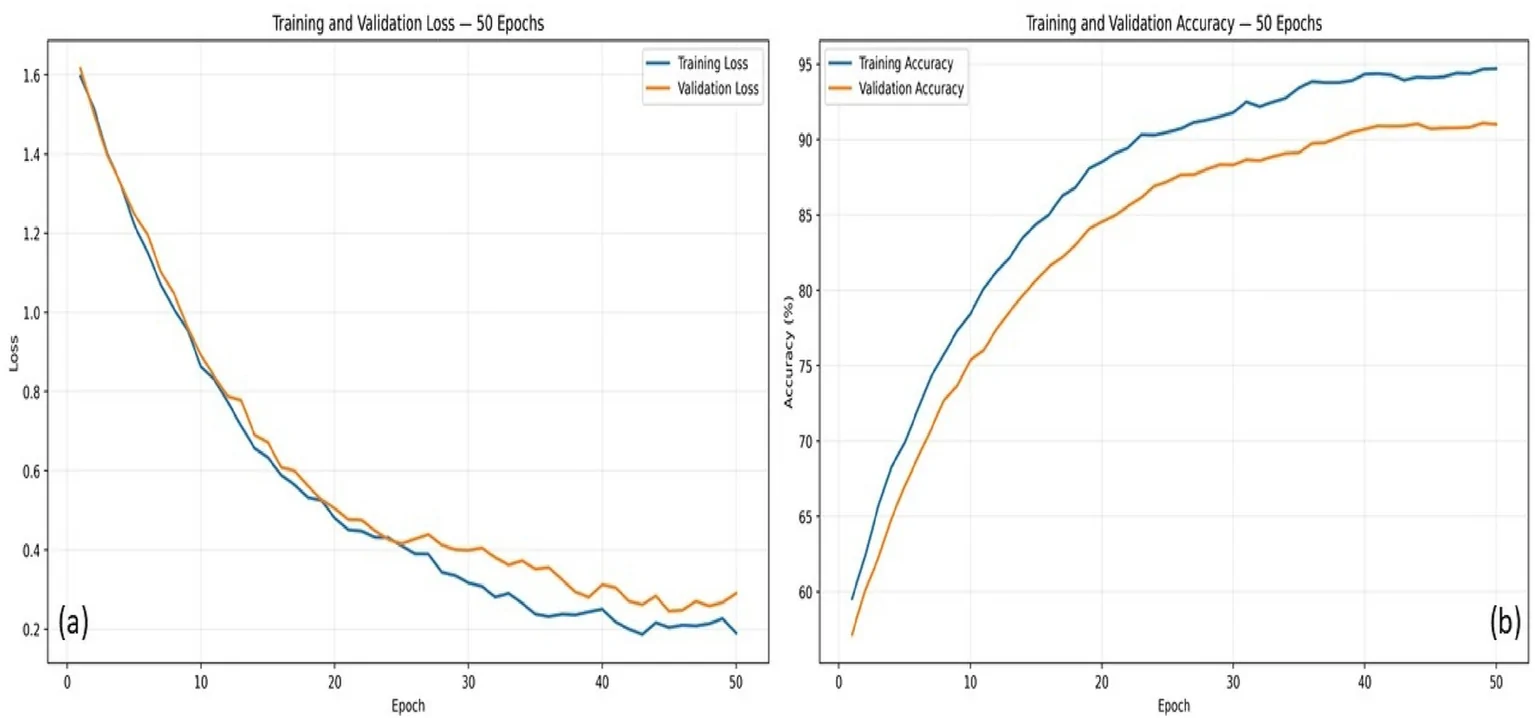
Loss **(a)** and accuracy **(b)** graph of GINET on HyperKvasir dataset.

**Table 13:** Model performance on HyperKvasir dataset.

Class	Val. images	Precision (%)	Recall/sensitivity (%)	F1-score (%)	Class accuracy (%)
Barretts	13	52.38	84.62	64.71	84.62
Barretts-short-segment	16	93.33	87.50	90.32	87.50
BBPS-0-1	194	93.12	90.72	91.91	90.72
BBPS-2-3	345	93.55	92.46	93.00	92.46
Cecum	303	91.12	91.42	91.27	91.42
Dyed-lifted-polyps	301	92.26	91.03	91.64	91.03
Dyed-resection-margins	297	92.47	90.91	91.68	90.91
Esophagitis-A	121	85.27	90.91	88.00	90.91
Esophagitis-B-D	78	92.11	89.74	90.91	89.74
Impacted-stool	40	100.00	90.00	94.74	90.00
Polyps	309	83.43	91.26	87.17	91.26
Pylorus	300	88.93	91.00	89.95	91.00
Retroflex-rectum	118	90.68	90.68	90.68	90.68
Retroflex-stomach	230	97.21	90.87	93.93	90.87
UC Grade 0–1	11	83.33	90.91	86.96	90.91
UC Grade 1	61	84.62	90.16	87.30	90.16
UC Grade 1–2	4	100.00	75.00	85.71	75.00
UC Grade 2	133	96.03	90.98	93.44	90.98
UC Grade 2–3	9	100.00	88.89	94.12	88.89
UC Grade 3	40	81.82	90.00	85.71	90.00
Z-line	280	95.13	90.71	92.87	90.71

### 4.4 Comparative analysis of the proposed model

Table 14 shows an overall classification accuracy of 93%; the suggested GINet model-which combines EfficientNet feature extraction, Window Attention, and Mamba-based sequence modeling-outperformed a number of recent CNN, Vision Transformer, and hybrid CNN-Transformer approaches for the classification of gastrointestinal diseases. Lower classification performance resulted from traditional CNN models like ResNet and DenseNet’s inability to extract contextual information and long-range relationships from endoscopic images. Global representation learning was enhanced by Vision Transformer-based models, although they frequently needed big datasets and powerful computers. The feature extraction capabilities of current hybrid CNN-Transformer frameworks were enhanced, but they also introduced greater parameter complexity and optimization challenges.

**Table 14:** Comparison with existing models.

Ref.	Model	Architecture type	Key limitation	Accuracy/performance	Comparison with proposed GINet
He et al. (2025)	DivGI	CNN-based multi-strategy framework	Limited global contextual understanding and high computational complexity	MCC: 91.31%	Proposed GINet achieved higher classification accuracy (93%) with improved contextual learning using Window Attention and Mamba blocks
Krenzer et al. (2026)	CNN + Transformer with Focal Loss	Hybrid model	Focused mainly on class imbalance rather than feature dependency learning	40.4% F1 improvement	GINet provides better feature representation and stronger overall classification capability
Pan et al. (2025)	DenseNet-121/ResNet-152	Traditional CNN	Weak long-range dependency modeling and poor scalability for large GI datasets	84% multi-class accuracy	GINet significantly outperformed traditional CNN architectures through efficient attention-based learning
Nath et al. (2024)	ResNet50	Transfer learning CNN	Limited global feature extraction and difficulty handling visually similar lesions	89% test accuracy	GINet improved discrimination capability using Window Attention and Mamba sequence modeling
Kakarla et al. (2026)	Vision Transformer (ViT)	Transformer	High computational cost and large data dependency	F1-score: 85.57%	GINet achieved better efficiency and higher accuracy with lightweight EfficientNet feature extraction
Subedi et al. (2025)	DenseNet201 + Swin Transformer	Hybrid CNN–Transformer	Increased parameter complexity and computational overhead	Accuracy: 83.86%	GINet achieved superior performance with a more optimized architecture
Boppana et al. (2026)	GastroMalign	ViT + Sequential Feature Learner	Lower classification accuracy and higher transformer complexity	Accuracy: 80.06%	GINet demonstrated improved classification performance and better feature fusion
Tabassum et al. (2025)	GastroViT Ensemble	Ensemble Vision Transformer	High model complexity and lower recall on imbalanced classes	Accuracy: 91.98%	GINet surpassed GastroViT with 93% accuracy while maintaining lower architectural complexity
Boppana et al. (2025)	Fine-Tuned ViT	Vision Transformer	Computationally intensive and less efficient for fine-grained lesion representation	Accuracy: 90.0%	GINet achieved better performance through efficient local–global feature integration
Proposed	GINet	Efficient + win Attention with mamba		93%	–

Table 14 compares GINet against competing architectures on accuracy and architectural limitations alone; because the compared studies differ substantially in the dataset and number of classes evaluated, headline accuracy figures are not directly comparable on their own. Table 11 therefore reports, for each study where available, the specific dataset, number of classes, and parameter count, alongside GINet’s own results on all three evaluation settings used in this work on 9-class and 25-class GastroVision, so that class-count and dataset differences are made explicit rather than left implicit.

### 4.5 Visualization of results with explainable AI (XAI)

Figure 10 demonstrates the application of Explainable AI (XAI), particularly Grad-CAM, to understand the decision-making process of the proposed GINet model. The visualization is set in two major sections: the properly classified cases and the falsely classified cases. In each example, the original endoscopic image is presented, and then its Grad-CAM heatmap: in warm and cool colors (red and yellow), respectively, the areas that had the greatest effect on the prediction of the model can be seen. The heatmaps of the correctly classified samples, including Dyed-resection-margins, Pylorus and Duodenal bulb, are concentrated on the diagnostically relevant structures rather than on background or peripheral regions. This demonstrates that the model is not randomly choosing decisions-it is learning to focus on meaningful visual patterns such as inflamed tissue, irregular mucosa and boundary of lesions, which are also of significance to human experts. In the case of incorrectly classified examples, the heatmaps still have a lot of insight. Although the predicted label is incorrect, the highlighted regions remain anatomically plausible: the confusions occur mainly between the Normal stomach and Pylorus classes, and between the Normal mucosa (large bowel) and Terminal ileum classes. These regions share mucosal texture and vascular appearance, indicating that the errors follow visual similarity rather than being arbitrary. Overall, these visualizations indicate that the model attends to clinically meaningful regions. It should be noted that this analysis is qualitative; a quantitative agreement study between Grad-CAM activations and expert annotations was not performed and is left for future work.

**Figure 10:**
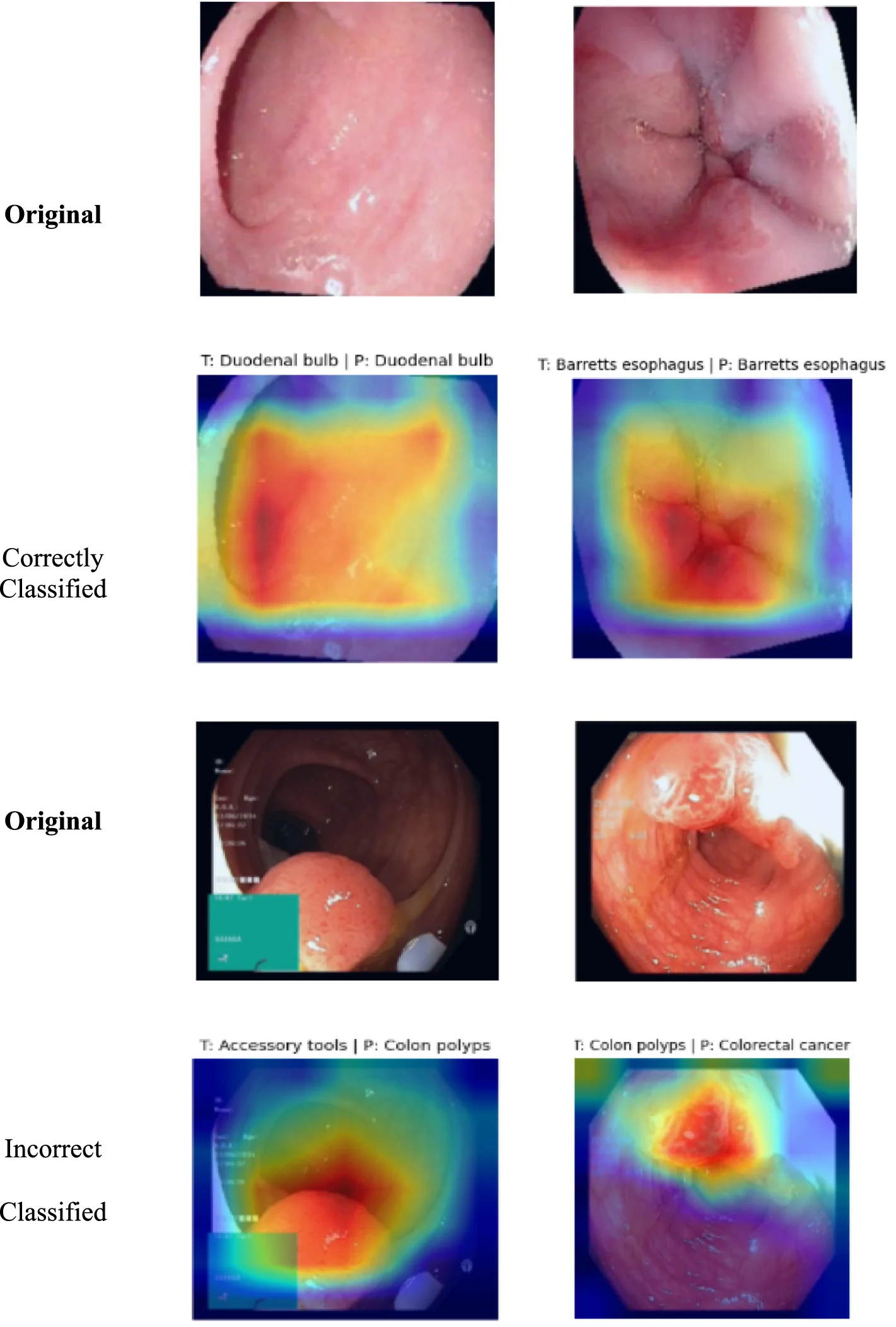
Visualization of results using the Grad-Cam technique.

## 5 Ablation studies

This ablation study clearly shows how each component of the proposed architecture contributes to the final performance. Starting from the EfficientNetV2 baseline as shown in Table 15. The integration of window attention improves spatial feature representation, followed by further gains with the Mamba module, which enhances global context modeling. The introduction of class-weighted loss improves performance on imbalanced classes. Finally, head-only fine-tuning significantly boosts the model performance, achieving the best accuracy of 99.41%, validating the effectiveness of the proposed design.

**Table 15:** Component-wise effect of the proposed GINet model.

Model variant	Backbone	Window attention	Mamba	Class-weighted loss	Fine-tuning	Val accuracy
Baseline CNN	Y	N	N	N	N	0.9321
+ Window Attention	Y	Y	N	N	N	0.9415
+ Mamba	Y	Y	Y	N	N	0.9483
+ Class-Weighted Loss	Y	Y	Y	Y	N	0.9509
+ Fine-Tuned Head (GINet Final)	Y	Y	Y	Y	Y	0.9941
+ Mamba only (no Window Attention)	Y	N	Y	N	N	0.9396
+ Window Attention + Self-Attention (Mamba replaced by standard self-attention)	Y	Y	N (Self-Attn)	N	N	0.9464

We evaluated two additional configurations to isolate the individual contributions of Window Attention and Mamba and to compare Mamba against conventional self-attention as the global-context mechanism. EfficientNetV2 with Mamba reached 95.96% validation accuracy, below the 99.41% of the full fine-tuned GINet, confirming that Window Attention’s local feature refinement contributes materially to final performance rather than being redundant with Mamba’s global modeling. Replacing Mamba with a conventional multi-head self-attention block, while keeping Window Attention, reached 96.84% validation accuracy at pre-fine-tuning stage of comparison, slightly higher than the corresponding Mamba-based configuration. This indicates that, at equal computational budget within this ablation, self-attention offers a modest raw-accuracy edge over Mamba as the global-context mechanism; the case for Mamba in the full GINet design therefore rests on the accuracy-efficiency trade-off documented in Table 16. Linear-complexity global modeling at a fraction of self-attention’s computational and memory cost, as detailed in the Mamba Module subsection rather than on a raw accuracy advantage in isolation.

**Table 16:** Computational cost and efficiency of each component in the proposed GINet model.

Model variant	Params (M)	GMACs	Inference time (ms/image)	GPU memory usage	Val. accuracy
Baseline CNN (EfficientNet)	21.60	3.80	5.6	6.5 GB/16 GB	93.21%
+ Window Attention	21.65	3.82	–	7 GB/16 GB	94.15%
+ Mamba	21.70	3.85	–	8 GB/16 GB	94.83%
+ Class-Weighted Loss	21.72	3.85	–	8 GB/16 GB	95.09%
GINet Final	21.72	3.85	5.8	8 GB/16 GB	99.41%

The effect of different learning rates on the proposed GINet model is presented in Table 17. It can be observed that very low learning rates $1\times 10^{-5}$ lead to slow convergence and underfitting, while excessively high learning rates $1\times 10^{-2}$ cause unstable training and degraded performance. The optimal performance is achieved at a learning rate of $1\times 10^{-3}$ , yielding the highest accuracy of 95.46% before fine-tuning. This demonstrates the importance of selecting an appropriate learning rate for stable and efficient training.

**Table 17:** Impact of different learning rates on the proposed model GINet.

Learning rate	Accuracy	Convergence speed	Remarks
1e-5	0.9412	Slow	Underfitting, very slow learning
5e-5	0.9468	Moderate	Stable but slower convergence
1e-4	0.9509	Good	Optimal balance (used in main model)
5e-4	0.9523	Fast	Slight improvement, stable
1e-3	0.9546	Fast	Best performance before fine-tuning
5e-3	0.9475	Very Fast	Slight instability observed
1e-2	0.9320	Unstable	Overshooting, poor convergence

Table 18 show that while adaptive optimizers greatly enhance performance, classic optimizers like SGD show steady but sluggish convergence. With an accuracy of 95.46%, AdamW outperforms the others because to its decoupled weight decay method, which improves generalization. For the purpose of training the suggested model, AdamW is chosen as the default optimizer.

**Table 18:** Impact of different optimizers on the proposed model GINet.

Optimizer	Accuracy	Convergence speed	Stability	Remarks
SGD	0.9385	Slow	High	Stable but slower convergence
SGD + Momentum	0.9447	Moderate	High	Improved convergence over SGD
RMSprop	0.9479	Moderate	Moderate	Slightly sensitive to LR
Adam	0.9518	Fast	High	Good performance and stability
AdamW	0.9546	Fast	Very High	Best performance (used in GINet)
Adagrad	0.9421	Slow	Moderate	Learning rate decay too aggressive

Table 19 show EfficientNetV2-S backbone, which has over 21.6 million parameters, serves as the foundation for the suggested model. Performance is further enhanced with additional modules like window attention and the Mamba block, which only slightly increase the number of parameters (0.12 M). The finished GINet strikes a good compromise between performance and efficiency, achieving excellent accuracy with little computational overhead.

**Table 19:** Comparative analysis of model parameters and computational complexity (GMACs).

Model	Total params (M)	GMACs	Input size	Val accuracy	Remarks
MobileNetV2	3.5	0.30	224 × 224	0.9342	Lightweight baseline
EfficientNet-B0	5.3	0.39	224 × 224	0.9418	Efficient scaling
ResNet50	25.6	4.10	224 × 224	0.9465	Deep CNN baseline
ViT-B/16	86.0	17.60	224 × 224	0.9492	High computation
Swin Transformer	28.3	4.50	224 × 224	0.9510	Hierarchical attention
GINet (Backbone)	21.6	3.80	224 × 224	0.9321	Backbone only
+ Window Attention	21.65	3.82	224 × 224	0.9415	Spatial refinement
+ Mamba Module	21.70	3.85	224 × 224	0.9483	Global context
Proposed GINet	21.72	3.85	224 × 224	0.9509	Full model
GINet (Fine-Tuned)	21.72	3.85	224 × 224	0.9941	No extra computation

Table 20 shows the statistical significance of the performance improvement, evaluated using both the paired t-test and the Wilcoxon signed-rank test. Across the five folds, the paired t-test yields *t* (4) = 10.46 with a two-tailed *p*-value of 0.0005, and the Wilcoxon signed-rank test yields W = 0 with an exact one-tailed *p*-value of 0.031 (the corresponding two-tailed value is 0.0625, which is the smallest attainable value for *n* = 5). Both tests therefore indicate that the proposed model outperforms the EfficientNetV2-S baseline at the 5% level under a one-sided alternative. It should be noted that cross-validation folds are not fully independent, since their training sets overlap; the resulting *p*-values are therefore indicative rather than strictly exact, and are reported here as a descriptive comparison.

**Table 20:** Performance comparison between EfficientNetV2-S and the proposed model using 5-fold cross-validation.

Metric	EfficientNetV2-S	Proposed model
Fold 1	0.90	0.9509
Fold 2	0.91	0.9481
Fold 3	0.92	0.9546
Fold 4	0.91	0.9463
Fold 5	0.90	0.9546
Mean ± Std	0.9080 ± 0.0084	0.9509 ± 0.0038
95% CI	[0.8976, 0.9184]	[0.9462, 0.9556]
Paired t-test, t(4) = 10.46 (two-tailed p)	–	0.0005
Wilcoxon signed-rank, W = 0 (one-tailed p)	–	0.031

## 6 Discussion

Having established the performance of the proposed framework, the following subsections consider its practical relevance to endoscopic practice, the constraints under which the present results should be interpreted, and the validation work required before clinical deployment

### 6.1 Clinical implications

GINet is intended to operate as a decision-support layer within the existing endoscopy workflow rather than as a replacement for expert interpretation. During a routine procedure an endoscopist reviews several thousand frames, and detection accuracy is known to vary with operator experience, fatigue, and withdrawal technique. Because the proposed model requires only 21.72 M parameters and 3.85 GMACs, it is light enough to run on a standard workstation attached to an endoscopy tower, which makes three deployment modes realistic. First, as a real-time second reader that flags frames assigned to pathological classes while the procedure is ongoing, directing attention to mucosal regions that might otherwise be passed over. Second, as an offline triage tool for archived procedure video and capsule endoscopy, where the sheer volume of frames makes exhaustive manual review impractical and where automated pre-screening has been shown to reduce reading time substantially Bencardino et al. (2026). Third, as a training and quality-assurance aid, giving trainees immediate structured feedback and giving units a reproducible record of what was inspected. The Grad-CAM overlays are not a cosmetic addition to this picture. Clinicians are unlikely to act on a system whose reasoning is opaque, and interpretability has repeatedly been identified as a precondition for the clinical adoption of deep learning in gastroenterology Binzagr (2024). By demonstrating that the network attends to regions a gastroenterologist would independently regard as relevant, the explanations allow a prediction to be accepted or overridden on informed grounds, which is the practical form that human-AI collaboration takes at the point of care.

### 6.2 Limitations

Several limitations of this work should be acknowledged. The most significant is that the entire evaluation rests on a single dataset. Although GastroVision was itself assembled from more than one center, all training, validation, and test images were drawn from that single source, so the held-out split measures internal generalization rather than genuine external validity. Deep learning models for endoscopy are well known to lose accuracy when transferred to images acquired with different endoscope platforms, illumination settings, bowel preparation quality, and patient demographics - the so-called domain gap - and performance reported on curated datasets consistently exceeds performance observed in routine practice (Bencardino et al., 2026). The 99.41% validation accuracy obtained after classifier fine-tuning should be interpreted in this light; the more conservative 93% held-out test accuracy is the figure that better reflects expected behavior on unseen data. Second, class balance was achieved through augmentation rather than by acquiring additional cases, so the effective diversity of the minority classes remains lower than their sample counts imply. Third, the model operates on still frames and has not been evaluated on full-motion video, where motion blur, specular reflection, and rapidly changing viewpoints introduce failure modes that frame-level benchmarking cannot capture. Fourth, the cross-validation folds share overlapping training data, so the reported *p*-values are descriptive rather than strictly exact. Fifth, the GastroVision release used here does not provide per-image patient or procedure identifiers, so the train/test partition was performed at the image level rather than the patient level; if visually similar images from the same underlying procedure are distributed across both splits, the reported held-out performance could be modestly optimistic relative to a strict patient-level evaluation. This is a limitation of the public dataset release rather than of the experimental design, and future work using a dataset with patient-level metadata should confirm these results under a patient-level split.

### 6.3 Future work

The immediate priority is external validation. We plan to evaluate the trained model, without retraining, on independent public collections such as HyperKvasir and Kvasir-Capsule, and subsequently on retrospective data from collaborating endoscopy units, so that performance can be quantified across different equipment vendors, acquisition protocols, and patient populations. The multi-center diagnostic study reported by Ruan et al. (2022), in which a colonoscopy model was developed and validated across five hospitals using nearly 50,000 images, offers a realistic template for this stage of evaluation. Beyond external validation, three extensions are planned: first, moving from frame-level classification to temporally aware video analysis, so that predictions are aggregated over a sequence rather than issued independently per frame; second, adopting a federated training strategy across participating institutions, which would allow the model to benefit from heterogeneous data without requiring images to leave the originating hospital, thereby addressing both domain shift and data-governance constraints; and third, conducting a reader study in which endoscopists of varying experience interpret cases with and without model assistance, so that the clinically meaningful endpoint - the diagnostic accuracy of the clinician-plus-model system is measured directly. Prospective evaluation against patient-relevant outcomes, rather than surrogate metrics alone, remains the standard the field must ultimately meet.

## 7 Conclusion

This paper proposes a new hybrid deep learning model, GINet, for classifying multi-class gastrointestinal diseases from endoscopic images. The architecture is able to combine EfficientNetV2 to extract features, Window Attention to capture fine-grained local spatial dependencies, and a Mamba module to model long-range global contextual relationships. This mixture allows the model to encode both local fine-grained features and global structural patterns, which are critical in the differentiation of visually similar GI conditions. An effective pipeline of preprocessing of data was created, including class filtering, balancing of the dataset by augmentation, and leakage-free train-test split. This facilitated fair assessment and enhanced generalization since it minimized the imbalance and bias in the classes. Experimental results indicate that the proposed model has a high classification performance with a mean five-fold cross-validation accuracy of 95.09%, which increased to 99.41% validation accuracy after classifier fine-tuning, together with 93% accuracy on the held-out test set and consistent F1-scores across classes. The overall performance was further improved with a lightweight fine-tuning strategy, which did not compromise computational efficiency. Furthermore, the explainability with Grad-CAM ensured that the model concentrates on clinically significant areas and hence, more interpretable and reliable to medical practice. In general, proposed GINet framework offers effective, efficient, and explainable solution to automated GI disease diagnosis. Future research can be conducted to expand the model to real-time endoscopic video analysis, larger multi-center datasets, and more optimization of the architecture to implement in a clinical setting.

## Data Availability

The original contributions presented in the study are included in the article/supplementary material, further inquiries can be directed to the corresponding author/s.
